# Enhanced Growth and Osteogenic Differentiation of Human Osteoblast-Like Cells on Boron-Doped Nanocrystalline Diamond Thin Films

**DOI:** 10.1371/journal.pone.0020943

**Published:** 2011-06-10

**Authors:** Lubica Grausova, Alexander Kromka, Zuzana Burdikova, Adam Eckhardt, Bohuslav Rezek, Jiri Vacik, Ken Haenen, Vera Lisa, Lucie Bacakova

**Affiliations:** 1 Institute of Physiology, Academy of Sciences of the Czech Republic, Prague, Czech Republic; 2 Institute of Physics, Academy of Sciences of the Czech Republic, Prague, Czech Republic; 3 Nuclear Physics Institute, Academy of Sciences of the Czech Republic, Prague, Czech Republic; 4 Institute for Materials Research (IMO), Hasselt University & Division IMOMEC, IMEC vzw, Diepenbeek, Belgium; University of Pennsylvania, United States of America

## Abstract

Intrinsic nanocrystalline diamond (NCD) films have been proven to be promising substrates for the adhesion, growth and osteogenic differentiation of bone-derived cells. To understand the role of various degrees of doping (semiconducting to metallic-like), the NCD films were deposited on silicon substrates by a microwave plasma-enhanced CVD process and their boron doping was achieved by adding trimethylboron to the CH_4_:H_2_ gas mixture, the B∶C ratio was 133, 1000 and 6700 ppm. The room temperature electrical resistivity of the films decreased from >10 MΩ (undoped films) to 55 kΩ, 0.6 kΩ, and 0.3 kΩ (doped films with 133, 1000 and 6700 ppm of B, respectively). The increase in the number of human osteoblast-like MG 63 cells in 7-day-old cultures on NCD films was most apparent on the NCD films doped with 133 and 1000 ppm of B (153,000±14,000 and 152,000±10,000 cells/cm^2^, respectively, compared to 113,000±10,000 cells/cm^2^ on undoped NCD films). As measured by ELISA per mg of total protein, the cells on NCD with 133 and 1000 ppm of B also contained the highest concentrations of collagen I and alkaline phosphatase, respectively. On the NCD films with 6700 ppm of B, the cells contained the highest concentration of focal adhesion protein vinculin, and the highest amount of collagen I was adsorbed. The concentration of osteocalcin also increased with increasing level of B doping. The cell viability on all tested NCD films was almost 100%. Measurements of the concentration of ICAM-1, i.e. an immunoglobuline adhesion molecule binding inflammatory cells, suggested that the cells on the NCD films did not undergo significant immune activation. Thus, the potential of NCD films for bone tissue regeneration can be further enhanced and tailored by B doping and that B doping up to metallic-like levels is not detrimental for cells.

## Introduction

Nanostructured materials, i.e. materials characterized by dimensions of less than 100 nanometres, are promising for a wide range of advanced technologies, including nanomedicine. In this novel interdisciplinary scientific field, nanostructured materials are developed or applied as carriers for targeted drug and gene delivery, as tracers for bioimaging, tools for nanoscale surgery, components of nanoelectronic biosensors, and also as cell carriers for tissue engineering, i.e. for constructing bioartificial replacements for irreversibly damaged tissues and organs.

Artificial materials currently used for constructing body implants, characterized by microscale topography of the cell-material interface, often do not evoke proper cellular responses needed for integrating them with the surrounding tissue and for tissue regeneration. The cells typically studied on these materials, i.e. anchorage-dependent mammalian cells of various tissues and organs, including the bone, adhere with spreading across usually tens of µm (for a review, see [1]). Thus, irregularities on the same scale can hamper appropriate spreading of the cells. The cells have to bridge the irregularities or to spread only in a limited space in the grooves among the prominences, which reduces the cell-substratum contact area, proliferation activity, viability and functioning of the cells, e.g., the activity of alkaline phosphatase in osteoblasts, necessary for bone tissue mineralization [2]–[5].

On the other hand, nanostructured materials imitate the nanoscale architecture of natural tissue components, such as extracellular matrix (ECM) and cell membrane with cell adhesion receptors. In addition, the ECM molecules, such as vitronectin, fibronectin, collagen or laminin, which mediate the cell adhesion on artificial materials, and are adsorbed spontaneously to the material surfaces, are attached in advantageous geometrical conformations, which enable good contact between specific bioactive sites in the ECM molecules (e.g., oligopeptidic ligands for cell adhesion receptors) and cell adhesion receptors (e.g., integrins) [6]–[8]. Thus, nanostructured materials are usually considered to be more promising than “classical” microstructured substrates for tissue engineering.

Nanostructured surfaces can be fabricated using various approaches, such as lithographic methods [9], [10], self-assembly processes [10], [11], treatment of polymers using plasma, ultraviolet light or ion irradiation [12], [13], etching of polymers using acids or hydroxides [6], creation of nanofibrous membranes using an electrospinning technique [14], and immobilization of biomolecules, such as extracelular matrix (ECM) molecules [15] or synthetic ECM-derived oligopeptidic ligands for cell adhesion receptors [16], on the material surface. Another important technique for creating nanostructured surfaces is deposition of nanoparticles on the material surface. For example, these nanoparticles include ceramic nanocrystals, such as hydroxyapatite [17], nanophase metals [8], [12], [18] and carbon nanofibres, nanotubes and fullerenes [7], [19]. Nanoparticles can also be admixed in a polymeric matrix designed for constructing three-dimensional porous or fibrous scaffolds for bone tissue reconstruction, in order to improve not only the mechanical properties but also the osteoconductive properties of the material [20].

However, all the nanostructured materials mentioned here may have some drawbacks, e.g., possible retention of chemicals in the polymers leading to cytotoxic action, brittleness of ceramics, release of cytotoxic and immunogenic ions from metallic materials (for a review, see [21]) as well as the potential cytotoxicity of fullerenes and carbon nanotubes (for a review, see [22]). Among all carbon materials, which are often presented as one of the most promising and natural forms, only nanocrystalline diamond (NCD) has shown excellent biocompatibility and no cytotoxicity *in vitro* and *in vivo*, and thus it seems to be the most advantageous material for biomedical applications (for a review, see [22], [23]). Other remarkable properties of NCD, such as high hardness, high wear, heat and chemical resistance, low friction coefficient and good electrical properties, also enable its application not only in electronics and optics, but also in biotechnologies and medicine, particularly in hard tissue surgery, e. g. as a mechanically and chemically resistant coating for bone and dental implants. This coating can be expected to prevent the release of ions and particles from the underlying bulk material, and also to improve integration of the implant with the surrounding bone tissue. In our earlier studies and in studies by other authors, NCD has proven itself as an excellent substrate for adhesion, growth, metabolic activity and phenotypic maturation of several cell types *in vitro*, including osteogenic cells [19], [24]–[28]. In studies *in vivo*, diamond layers, deposited by a microwave plasma chemical vapour deposition method (MW CVD) on Ti-6AI-4V probes and implanted into a rabbit femur, showed very high bonding strength to the metal base as well as to the surrounding bone tissue, and prevented material corrosion [29]. Nanocrystalline and multilayer diamond thin films were also used for coating the heads and cups of an artificial temporomandibular joint made of Ti-6AI-4V alloy [30]. Another advantageous property of NCD, useful for its biomedical applications, is its ability to bind various biological molecules, which can be utilized for detecting, separating and purifying these molecules, e.g. for detecting proteins diluted in solutions by MALDI-TOF mass spectrometry [31] or immobilization of antibodies for sensor application [32].

Another important feature of a cell adhesion substrate, promoting its colonization with cells, is its electrical charge and conductivity, even without active stimulation of the cells with an electric current and an electromagnetic field. For example, vascular endothelial cells, vascular smooth muscle cells and bone marrow stromal cells grew better on positively charged surfaces than on electroneutral polymeric surfaces [33]–[35]. A negative charge has also been reported to have favourable effects on cells: for example, rabbit chondrocytes in cultures on negatively charged hydrogels increased their production of collagen II [36].

Similarly, vascular smooth muscle cells adhered and grew better on synthetic polymers which were rendered electrically conductive by chemical doping with carbon black or by creating long carbon chains with conjugated double bonds after ion irradiation [37], [38].

It is well known that p-type electrical conductivity of NCD films can be achieved by boron doping [39]. Boron-doped NCD films have been applied in electronics and sensorics (for a review, see [40]) and for constructing cell microarrays on surfaces patterned with various chemical functional groups [41]. However, little is known about the direct influence of boron-doped NCD films on the adhesion, growth, differentiation and function of osteogenic cells, and thus on their potential use as a substrate for bone tissue regeneration. In our earlier study performed on boron-doped NCD films deposited on glass substrates [42], human osteoblast-like MG 63 cells in cultures on these films achieved higher final cell population densities than on uncoated glass, and also stained more intensively against osteocalcin, a calcium-binding non-collagenous ECM protein and an important marker of osteogenic cell differentiation. However, in that study [42], only one concentration of B (3000 ppm) was used for the cell experiments, and osteocalcin has not been biochemically quantified.

Therefore, in the present study, we have made a more systematic investigation of the adhesion, growth, viability, phenotypic maturation and potential immune activation of human osteoblast-like MG 63 cells cultured on NCD films doped with three distinctively different concentrations of boron (133, 1000 and 6700 ppm). These concentrations were chosen in order to prepare the following representative groups of samples: (i) low doped semiconducting samples (133 ppm of B), (ii) medium, commonly doped semiconducting samples (1000 ppm of B), and (iii) NCD films with metallic-like quality (i.e. boundary to overdoped films) achieved by the highest B doping level (6700 ppm). The NCD films were grown and characterized on silicon; this methodology was developed in our earlier preliminary study [43]. We also provide a comparison with undoped NCD films on silicon and standard cell culture substrates, i.e., tissue culture polystyrene and glass.

## Results

### Physical and chemical properties of NCD films

**Figure 1:**
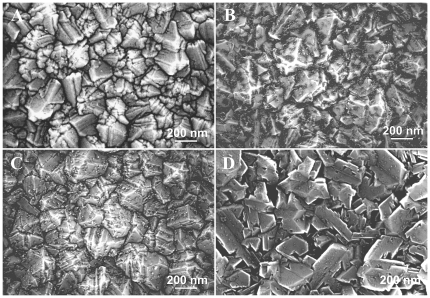
Surface morphology of NCD samples without boron doping (A), with boron 133 ppm (B), 1000 ppm (C) and 6700 ppm (D). Field emission scanning electron microscope (e_Line, Raith). Scale bar is 200 nm.

The surface morphology of the NCD samples grown on SiO_2_/Si substrates, as evaluated by field-emission scanning electron microscope (FESEM), is shown in **Fig. 1**. The intrinsic NCD film (i.e., 0 ppm boron doping) consisted of randomly oriented grains up to 200 nm in size (**Fig. 1A**). The root-mean-square (*rms*) surface roughness was 27±3 nm, as measured by AFM on a scan area of 1.5×1.5 µm^2^. Adding 133 ppm boron to the gas mixture did not change the main crystal character, but there were additional fine (nano-sized) crystallites (dark dots) distributed across the surface of larger crystals and filling the surface depressions predominantly (**Fig. 1B**). The *rms* surface roughness decreased slightly down to 20±3 nm due to these features. The increase in the boron concentration to 1000 ppm resulted in lower density and more uniform distribution of the fine crystallites across the surface (**Fig. 1C**). The *rms* roughness increased back to 27±4 nm. For the highest boron doping of 6700 ppm, flattened nanodiamond crystals with increased lateral size (400 nm) were observed. Fine crystallites appeared on these crystals in a low density compared to the other doping levels (**Fig. 1D**). The surface roughness increased slightly to 33±4 nm, most likely due to the larger size of the crystal grains.

**Figure 2:**
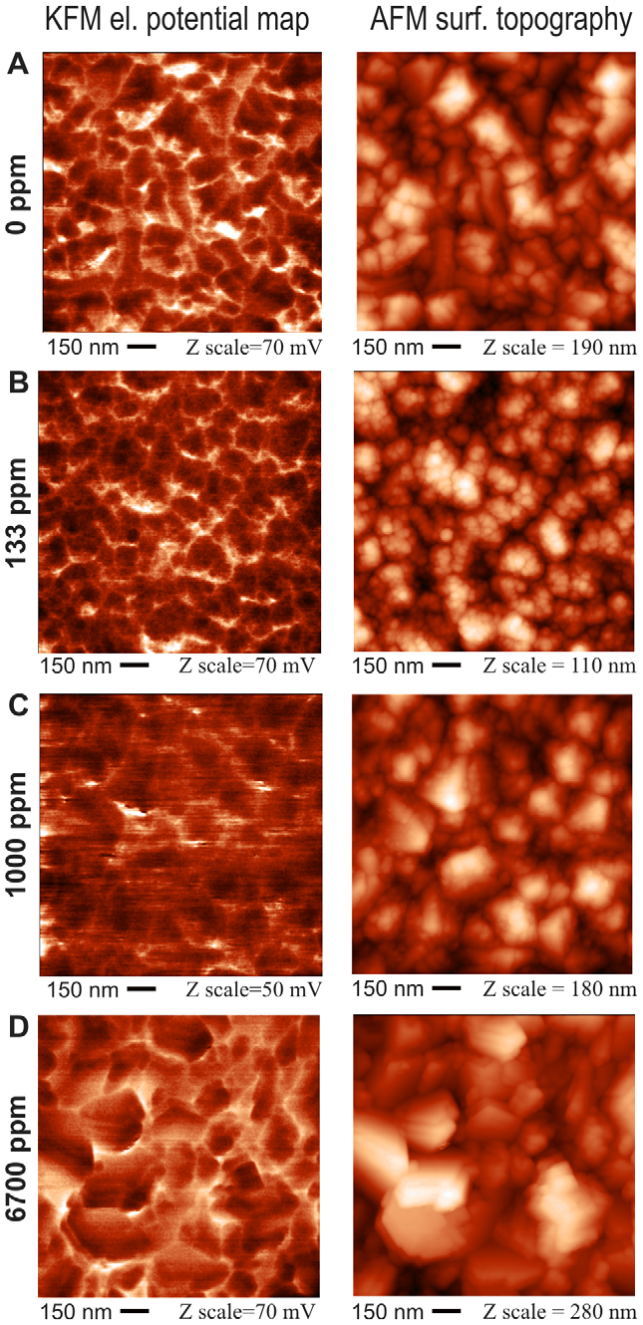
Microscopic images of surface potential variations as detected by Kelvin force microscopy (left column), and surface topography as detected by atomic force microscopy (right column) on NCD samples without boron doping (A) and with nominal boron doping of 133 ppm (B), 1000 ppm (C), and 6700 ppm (D). The AFM oscillation amplitude was 100 nm, the setpoint was 50%. The KFM lift height was 30 nm. The detection frequency was 75 kHz.

**Fig. 2** shows microscopic images of surface electrical potential variations on the above NCD samples, as detected by Kelvin force microscopy (KFM), scan area 1.5×1.5 µm^2^. The potential images are accompanied by AFM topography in the same place. In contrast to the FESEM images presented in **Fig. 1**, the AFM topography images show that AFM produces more rounded grain shapes. The reason is that the AFM resolution is limited by the tip dimensions. Due to the tip shape, the *rms* roughness values are generally slightly underestimated, yet a relative comparison can be made on the basis of a set of samples. The potential images show that the electrical potential is homogeneous on the grains within the whole employed range of boron doping (0–6700 ppm). The inter-grain (overall) fluctuations can be attributed mostly to the height variations of the sample, which may influence the capacitive coupling between the AFM/KFM tip and the surface [44]. This effect is expected to be similar for all samples, due to their similar morphology and *rms* roughness. Similar standard deviations of surface potentials (see **Table 1**) indicate that this is indeed the dominating effect. As shown in **Tab. 1**, the surface potentials on all samples *versus* a grounded Au electrode were between 40±4 mV to 96±6 mV. The highest surface potential (96±6 mV) was found on the 133 ppm sample. Positive potential values correspond to a higher surface work function or to a more negative surface charge. AFM in the vibrational regime was also used to detect a phase contrast, which can be used as an indication of surface quality [45]. In our case, very small variations of 2–4 degrees in the phase contrast are all within the error bar and indicate constant quality of the diamond surface in the whole sample series. The values of *rms* surface roughness, AFM phase contrast and relative surface potential of NCD films as obtained by KFM are summarized in **Table 1**.

**Table 1:** Dependence of the surface parameters of the NCD samples on the boron doping level.

Sample Type	B Doping Level	Roughness*rms*	Surface Potential*	AFM phase *rms*	Resistivity	Contact Angle
I. 0 ppm	N/A	27±3 nm^II, IV^	40±4 mV^II, III, IV^	2±1 deg^II^	>10 MΩ	17±6°^IV^
II. 133 ppm	2×10^19^ cm^−3^	20±3 nm^I, III, IV^	96±6 mV^I, III, IV^	4±1 deg^I^	55 kΩ	18±5°^IV^
III. 1000 ppm	1×10^20^ cm^−3^	27±4 nm^II, IV^	47±4 mV^I, II, IV^	3±1 deg	0.6 kΩ	20±5°^IV^
IV. 6700 ppm	6×10^20^ cm^−3^	33±4 nm^I, II, III^	42±5 mV^I, II, III^	3±1 deg	0.3 kΩ	14±4°^I, II, III^

*Values adjusted to the surface potential of gold.

For the NDP measurements, the accuracy can achieve 5% (which is the precision of the boron atoms in the etalon). However, the precision of the NDP technique also depends on other parameters, e.g. the stability of the neutron beam intensity, identical geometry of the etalon and the measured sample, etc. Realistically, the NDP data can be routinely measured with accuracy of 10% in our case.

For roughness, potential, phase and contact angle, the data is presented as Mean ± S.D. (Standard Deviation). In the case of roughness and AFM phase, each *rms* value was determined from 65 536 data points on each sample type. The mean and S.D. of *rms* values were calculated from 5 such measurements across the sample. In the case of surface potential, the mean and S.D. values were calculated from 65 536 measurements across each sample. The contact angle was calculated from fitting the curve of the water droplet, and the mean and S.D. values were calculated from 16 measurements for each sample type.

Statistical Analysis: ANOVA, Student-Newman-Keuls Method. Statistical significance: ^I, II, III, IV^: *p*≤0.05 compared to the group labelled with the same Roman number.

For the room temperature electrical resistivity measurements, the accuracy is better than 1%.

**Figure 3:**
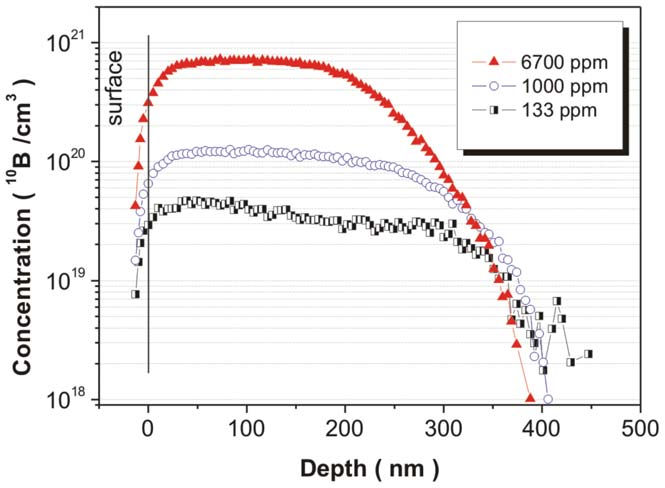
Neutron depth profiling of boron over doped NCD samples.

Neutron depth profiles of boron-doped NCD samples are shown in **Fig. 3**. The sample deposited at 133 ppm exhibited fluctuations in the boron content - in the first ≈200 nm (from surface to bulk), a slightly higher boron content was observed than that in the remaining part (depth from 200 to 400 nm). The boron concentration at the surface achieved a value of 2×10^19^ cm^−3^. The calculated full-width at the half-maximum (FWHM) for the boron profile was 314 nm. Medium doping (1000 ppm of B) led to increased boron content in the film up to 1×10^20^ cm^−3^ with an FWHM value of 282 nm. The highest boron doping (6700 ppm) resulted in the highest boron content of 6×10^20^ cm^−3^ and the lowest FWHM value of 221 nm. It should be noted that the data plotted to the left of the black line is not relevant; the line shows the detection limit of the measurement setup.

The nominally undoped sample (0 ppm) exhibited the highest resistivity >10 MΩ (the detection limit of our measurement setup). The introduction of low boron doping to 2×10^19^ cm^−3^ (133 ppm) resulted in a steep decrease in resistivity by three orders in magnitude down to approx. 55 kΩ. A further increase in boron to 1×10^20^ cm^−3^ (1000 ppm) decreased the resistivity to 0.6 kΩ. Finally, the samples doped by the highest boron level of 6×10^20^ cm^−3^ (6700 ppm) exhibited saturation of resistivity at 0.3 kΩ. It should be noted that this measurement was in good agreement with neutron depth profiling and thus, from an electrical point of view, the NCD samples that were used can be classified as highly resistive (0 ppm), semiconducting (133 or 1000 ppm), and metallic-like (6700 ppm).

The static water drop contact angle measurements revealed that the intrinsic NCD samples and all boron-doped samples were relatively highly hydrophilic (13–20°). The contact angle value increases slightly from 17° (nominally undoped sample) up to 20° for the boron doped sample 1000 ppm. For the highest boron doping level (6700 ppm), the value dropped to 13°.

The values of boron concentration, electrical resistivity, and water contact angle on the films are also summarized in **Table 1**, together with AFM and KFM data.

**Figure 4:**
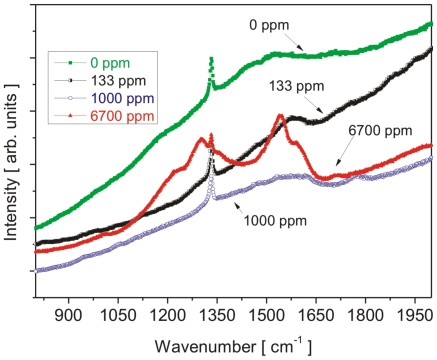
Raman spectra of undoped and boron-doped NCD samples.

Raman measurements confirmed the diamond character for all of the deposited films (**Fig. 4**). The position of the diamond peak remained within 1 cm^−1^ (i.e. well within the limits of accuracy) at a value of 1332 cm^−1^. The highly doped NCD films (6700 ppm) exhibited a remarkable change in the shape of the spectrum. The diamond peak was lowered and narrowed (a typical Fano-type lineshape appeared), and the graphite- and amorphous carbon-related bands at 1300 and 1540 cm^−1^ dominated the spectrum. In addition, a broad band appeared below 500 cm^−1^ and two new bands appeared centered at 1000 and 1200 cm^−1^. The development of new bands at high boron doping levels has also been observed before, and their origin was assigned to morphological changes (i.e., from micro- to nano-crystalline character) and to the non-diamond sp^2^ bonded carbon phases or conjugated double bonds [46], [47]. In our case, conjugated double bonds are most likely the dominating factor, as FESEM shows the largest grains on the 6700 ppm sample.

### Cell number, viability, spreading and proliferation

**Figure 5:**
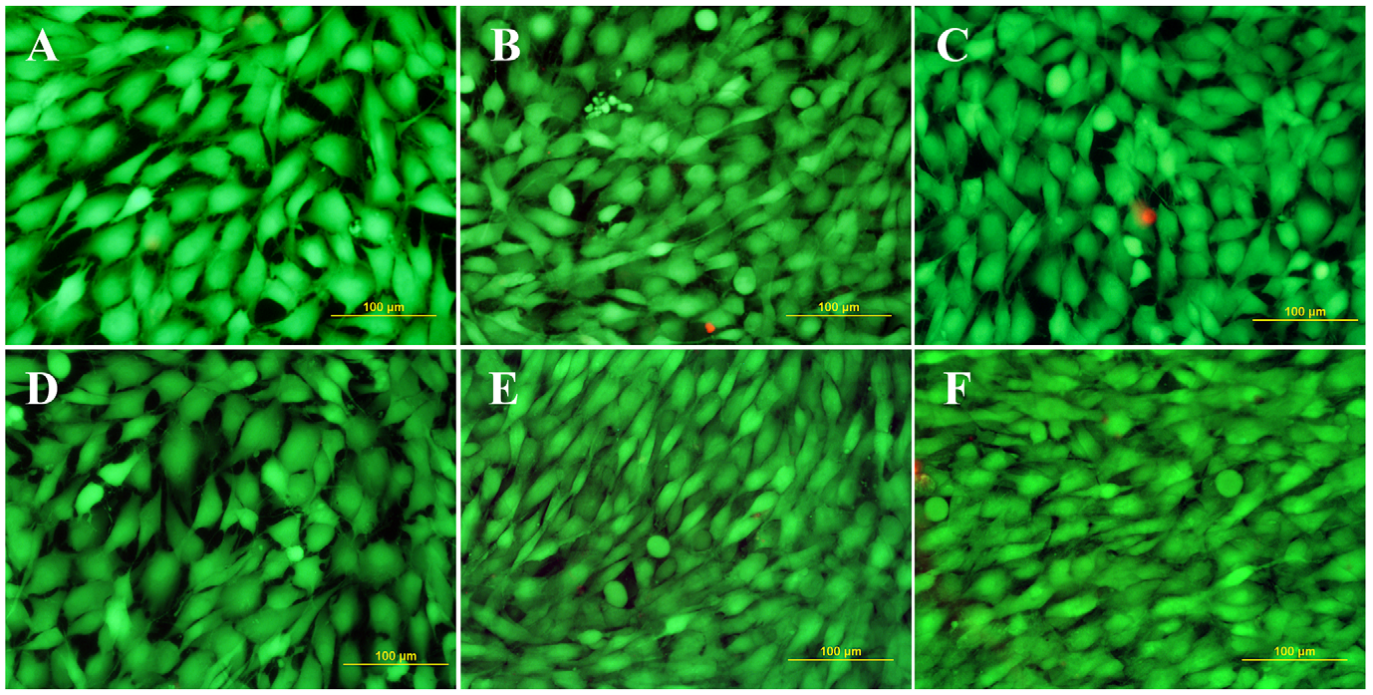
Fluorescence staining of MG 63 cells with a LIVE/DEAD viability/cytotoxicity kit (Invitrogen, Molecular Probes, U.S.A.) on day 7 after seeding on a microscopic glass coverslip (A) standard polystyrene cell culture dish (B), undoped NCD films (C), NCD films doped with boron in concentrations of 133 ppm (D) 1000 ppm (E) and 6700 ppm (F). Viable cells are stained in green with calcein, dead or damaged cells in red with ethidium homodimer-1. Olympus IX 51 epifluorescence microscope, DP 70 digital camera, obj. 20×, bar = 100 µm.

The cell number and the viability of cells on NCD surfaces were monitored on day 1, 3 and 7 after seeding. In evaluating the cell number, only viable cells were taken into account; however, the percentage of viable cells stained with calcein AM reached almost 100% on all tested samples, as indicated in **Fig. 5**.

**Figure 6:**
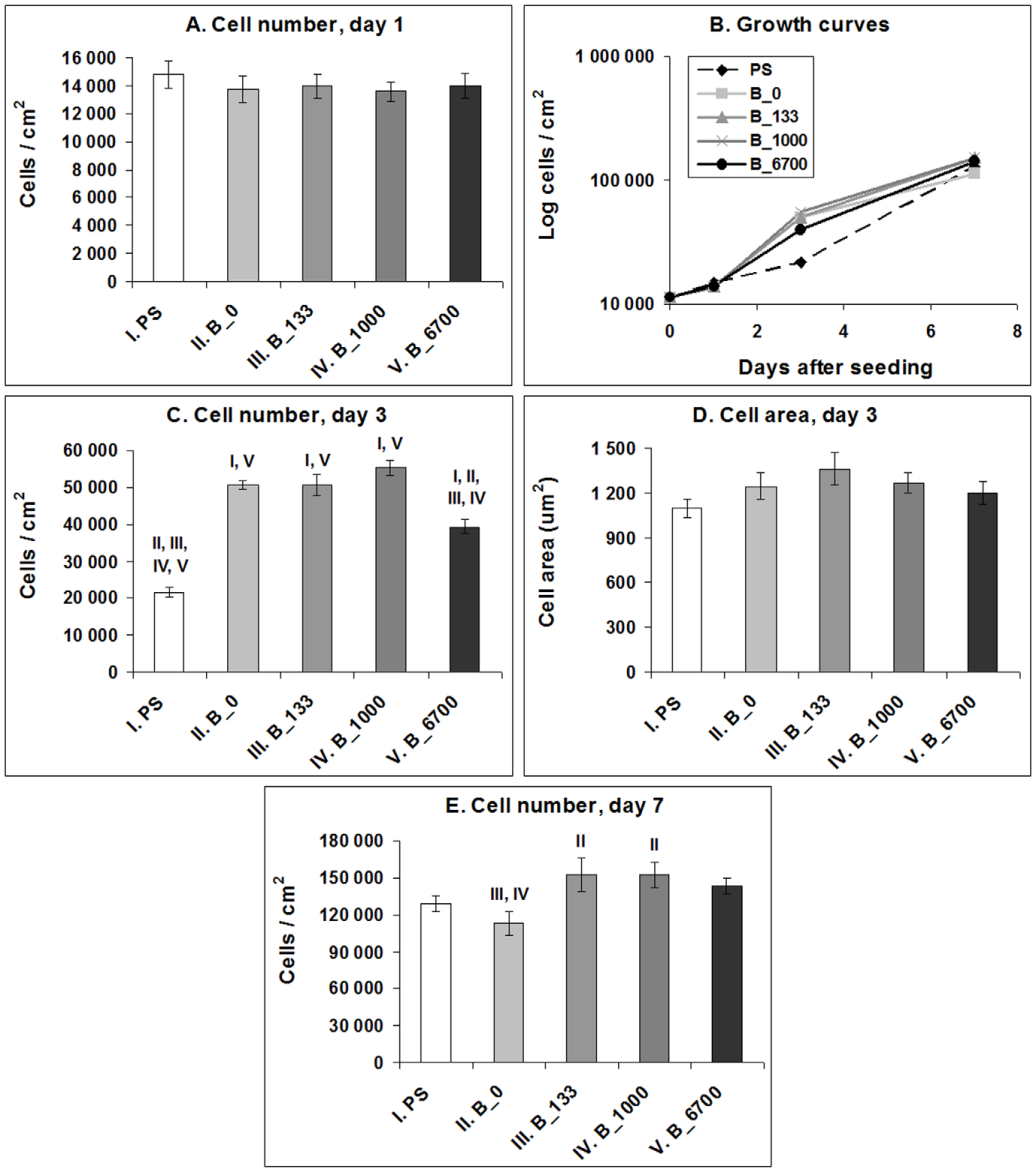
Number of MG 63 cells on day 1, 3 and 7 (A, C, E), their spreading area (D) and their growth dynamics (B) on a standard polystyrene cell culture dish (PS), undoped NCD films (B_0) and NCD films doped with 133, 1000 and 6700 ppm of boron (B_133, B_1000 and B_6700, respectively). Mean ± S.E.M. from 3 experiments; each included 32 microphotographs (day 1 and 3) and 18 measurements in a hemocytometer (day 7) per experimental group). ANOVA, Student-Newman-Keuls method. Statistical significance: I, II, III, IV, V: *p*≤0.05 compared to the group labelled with the same Roman number.

On day 1, the numbers of initially adhered cells on all boron-doped NCD films ranged from 13,600±700 to 14,000±900 cells/cm^2^) and were similar to the values found on the undoped samples (13,700±900 cells/cm^2^) and on standard polystyrene culture dishes (14,800±900 cells/cm^2^), which were used as a reference material (**Fig. 6A**).

**Figure 7:**
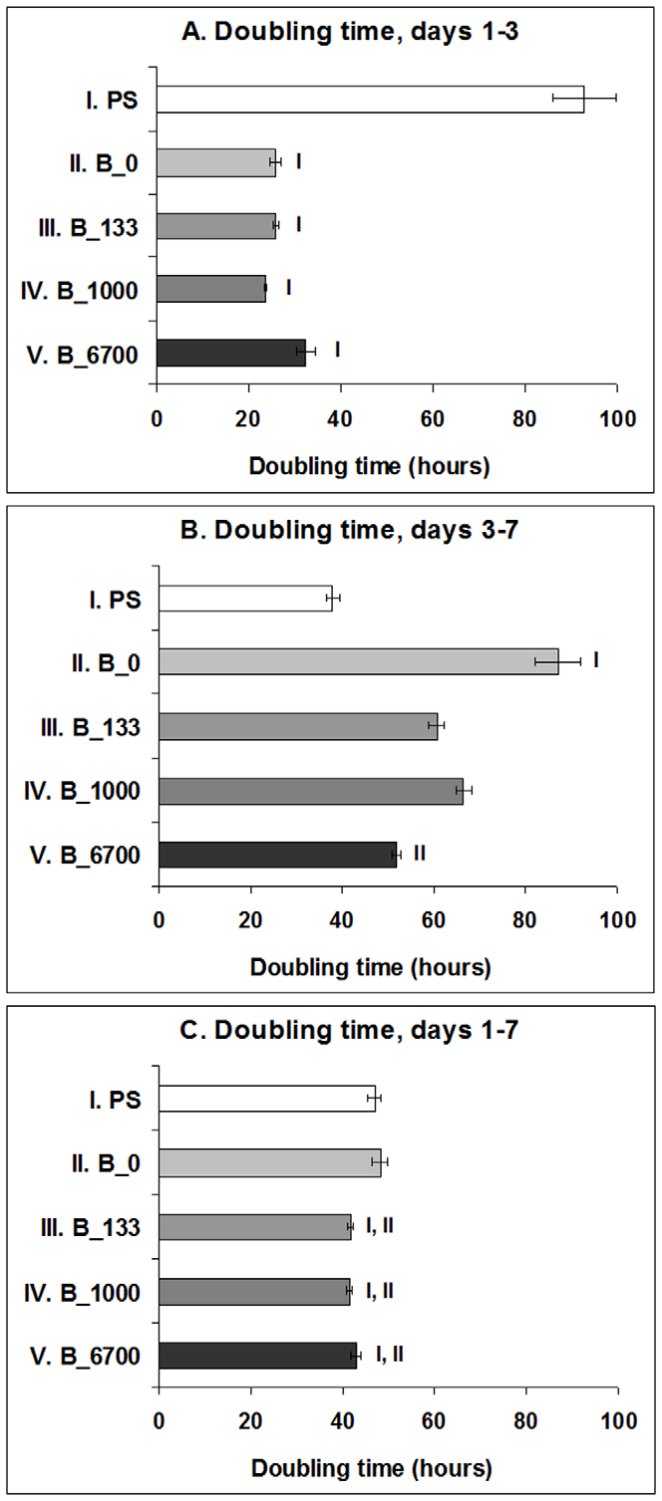
The cell population doubling time of MG 63 cells between days 1 and 3 (DT_1–3_), days 3 and 7 (DT_3–7_) and days 1 and 7 (DT_1–7_) after seeding on polystyrene culture dishes (PS) and NCD films doped with 0, 133, 1000 or 6700 ppm of boron. Mean ± S.E.M. from 3 experiments (in total, 9 measurements for each experimental group and time interval). ANOVA, Student-Newman-Keuls Method. Statistical significance: **^I, II, V^**: *p*≤0.05 compared to polystyrene, undoped NCD and NCD doped with 6700 ppm of B, respectively.

However, from day 1 to 3 after seeding, the cells on all tested NCD films entered the exponential phase of growth, whereas in the cells on the control polystyrene dishes, the proliferation started slowly, and the cells practically remained in the lag phase, as indicated by the shape of the growth curves (**Fig. 6B**). The population doubling time of the cells on the NCD films ranged approximately between 24 and 33 hours, while in the cells on polystyrene dishes the doubling time was on an average about 93 hours (**Fig. 7A**). As a result, on day 3 after seeding, the cell population density on all tested NCD films significantly exceeded (i.e., almost twice and more) the cell population on the control polystyrene dish (22,000±1,000 cells/cm^2^). The highest cell numbers (ranging from 51,000±1,000 cells/cm^2^ to 55,000±2,000 cells/cm^2^) were obtained on undoped NCD films and on films doped with 133 or 1000 ppm of B (**Fig. 6C**). On NCD films with 133 ppm of B, the cells also showed a tendency to be best spread, i.e. to adhere with the largest cell-material projected area, but this difference was not statistically significant (**Fig. 6D**). On the other hand, the cell number on NCD doped with 6700 ppm of B was relatively low (39,000±2,000 cells/cm^2^), and was significantly lower than the values on the other NCD films (**Fig. 6B**).

From day 3 to 7, the cells on all NCD surfaces slowed down their growth, especially on undoped NCD films, where they proliferated with a significantly longer doubling time than the cells on the control polystyrene dishes (**Fig. 7B**). As a result, the cells on polystyrene dishes achieved a cell population density (129,000±7,000 cells/cm^2^) which did not differ significantly from the densities on the other samples (from 113,000±10,000 cells/cm^2^ to 153,000±14,000 cells/cm^2^). Thus, on day 7 after seeding, the differences in cell numbers among the tested materials observed on day 3 partly disappeared. Nevertheless, the cell numbers on NCD films doped with boron in concentrations of 133 ppm (153,000±14,000 cells/cm^2^) and 1000 ppm (152,000±10,000 cells/cm^2^) still significantly exceeded the cell number on undoped NCD films (113,000±10,000 cells/cm^2^, **Fig. 6E**).

When the doubling time was calculated from day 1 to 7, it was significantly shorter on all boron-doped samples (from about 41 to 43 hours) than on polystyrene dishes and undoped NCD (47 and 48 hours, respectively (**Fig. 7C**).

### Distribution and concentration of molecules participating in cell adhesion, differentiation and immune activation

**Figure 8:**
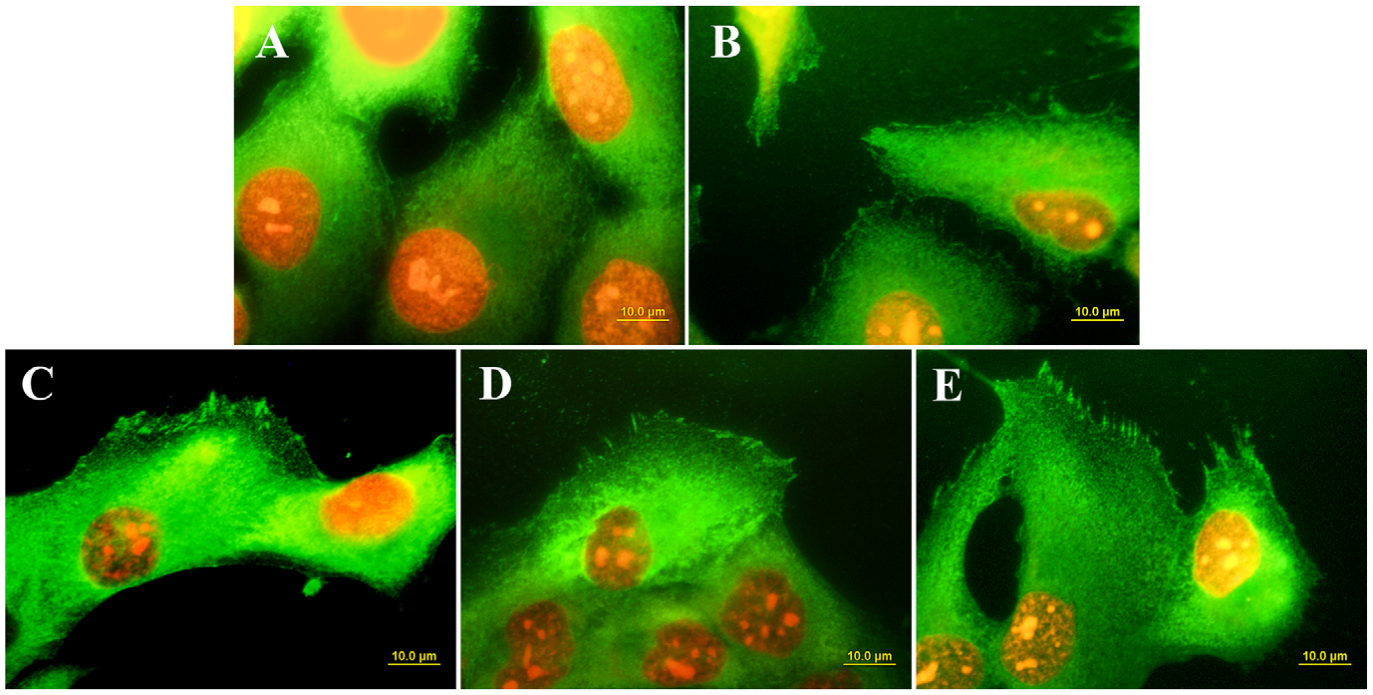
Immunofluorescence staining of talin in MG 63 cells on day 3 after seeding on microscopic glass coverslips (A), undoped NCD (B), NCD films doped with boron in concentrations of 133 ppm (C) 1000 ppm (D) and 6700 ppm (E). The cell nuclei are counterstained with propidium iodide. Olympus IX 51 epifluorescence microscope, DP 70 digital camera, obj. 100×, bar = 10 µm.

**Figure 9:**
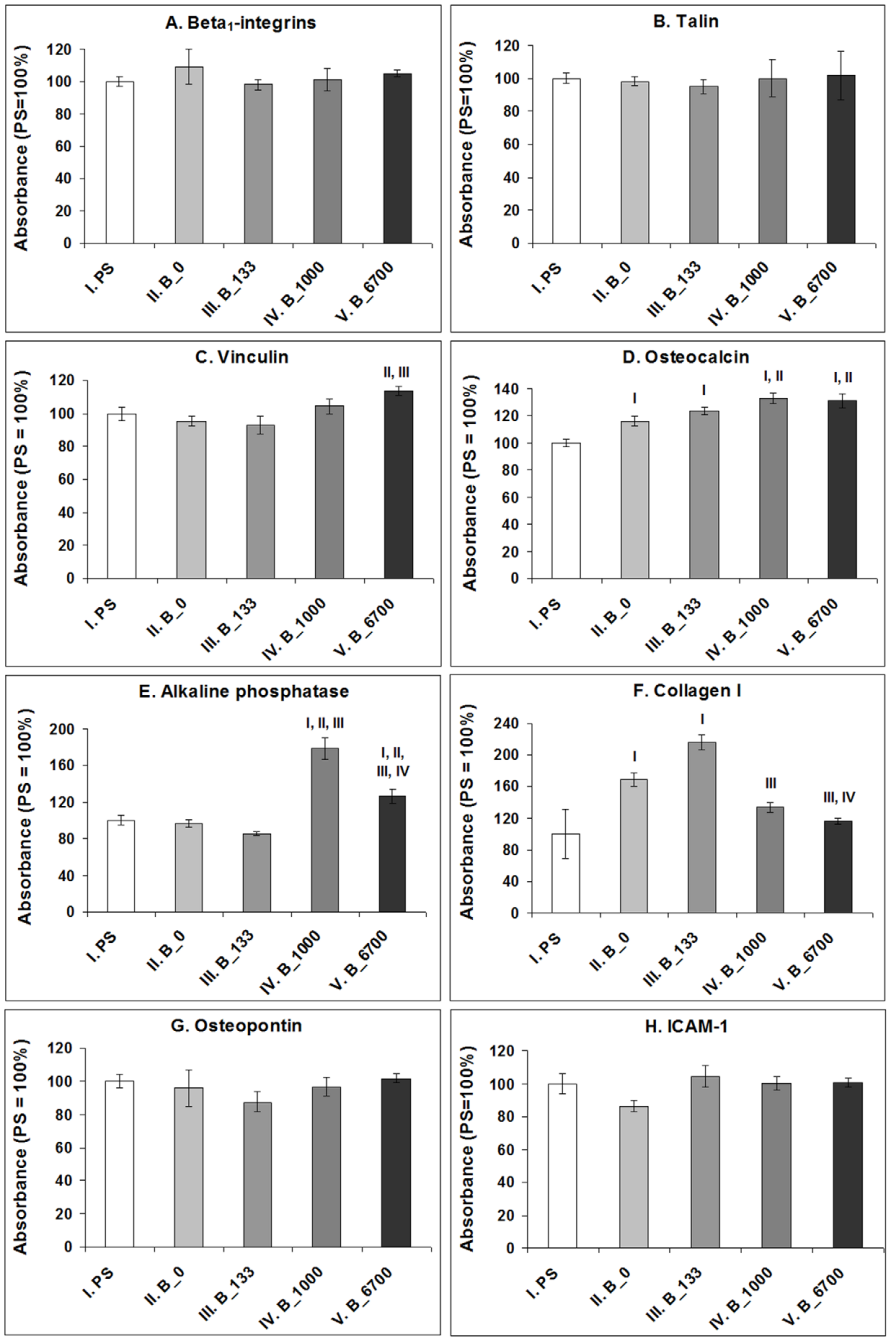
Concentration of β_1_-integrin adhesion receptors, integrin-associated focal adhesion proteins talin and vinculin, markers of osteogenic cell differentiation osteocalcin, alkaline phosphatase, collagen I and osteopontin, and immunoglobuline adhesion molecule ICAM-1, a marker of cell immune activation, in MG 63 cells on day 7 after seeding on polystyrene culture dishes (PS) and NCD films doped with 0, 133, 1000 or 6700 ppm of boron. Measured by ELISA per mg of protein. The absorbances are given as a percentage of the control values obtained on PS. Mean ± S.E.M. from 3 experiments (each included 12–16 measurements for each experimental group). ANOVA, Student-Newman-Keuls Method. Statistical significance: ^I, II, III, IV^: *p*≤0.05 compared to the group labelled with the same Roman number.

Immunofluorescence staining of talin, i.e. a protein of focal adhesion plaques associated with cell adhesion receptors, revealed that this protein was organized into large dash-like focal adhesion plaques in cells cultured on NCD films doped with boron, while in cells grown on undoped films and microscopic glass coverslips, talin was distributed more diffusely and formed only tiny and rather dot-like focal adhesion plaques (**Fig. 8**). As revealed by ELISA, the total concentration of talin (i.e., both bound and unbound in focal adhesion plaques) was similar in cells on all tested surfaces (**Fig. 9**). Also the concentrations of integrins with β_1_ chain, which involve receptors mainly for collagen, laminin and fibronectin, were similar in the cells on all tested films. However, the concentration of vinculin, another focal adhesion protein, was significantly higher in cells cultured on NCD with 6700 ppm of B than in cells on undoped NCD and NCD doped with 133 ppm of B (by 19% and 21%, respectively). Moreover, the concentration of osteocalcin (a calcium-binding ECM protein and an important marker of osteogenic cell differentiation) in cells cultured on all NCD films significantly exceeded (by 24 to 33%) the value in the cells on the control polystyrene culture dish. In addition, the concentrations of osteocalcin in cells on NCD with 1000 and 6700 ppm of B were significantly higher than the concentrations on undoped NCD films. Also the concentration of alkaline phosphatase, i.e., an enzyme participating in bone matrix mineralization, was higher (by 27 to 110%) in cells on NCD with 1000 and 6700 ppm of B than in cells on the other samples. The concentration of collagen I, an important component of bone matrix, reached the highest value in cells on NCD doped with 133 ppm of B (by 28 to 116% compared with the values on polystyrene and NCD with both higher B concentrations). On the other hand, the concentration of osteopontin, another marker of osteogenic cell differentiation, remained unchanged on the boron-doped samples (**Fig. 9**).

A favourable finding was that the concentration of ICAM-1, i.e. an immunoglobuline adhesion molecule which binds to β_2_-integrin adhesion receptors on inflammatory cells (e.g. leucocytes, monocytes, macrophages), was similar on cells on all tested surfaces, i.e. it was not higher in cells on undoped and boron-doped NCD films than in cells on standard cell culture polystyrene dishes (**Fig. 9**).

### Adsorption of collagen I to NCD films

**Figure 10:**
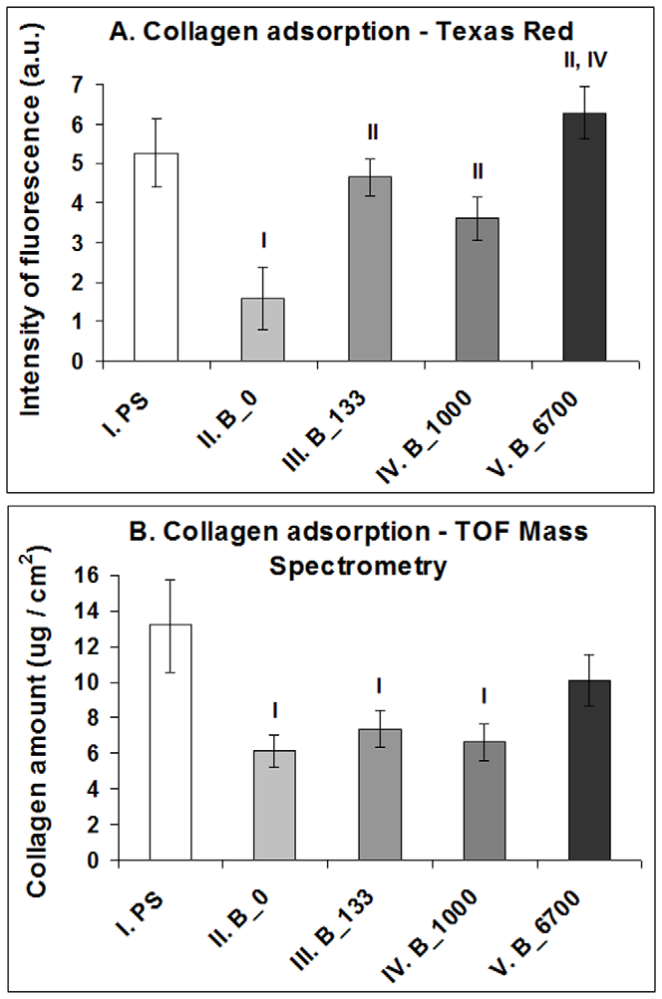
Adsorption of collagen I to NCD films with 0, 133, 1000 or 6700 ppm of boron and polystyrene (PS). **A**: Intensity of fluorescence after conjugation of collagen with Texas Red C_2_-Maleimide; **B**: Biochemical analysis using High Performance Liquid Chromatography (HPLC) combined with Time of Flight Mass Spectrometry (TOF-MS). Mean ± S.E.M from 11–13 measurements (**A**) or 9 measurements (**B**) for each experimental group. ANOVA, Student-Newman-Keuls Method. Statistical significance: ^I, II, III, IV^: *p*≤0.05 compared to the group labelled with the same Roman number.

The intensity of the fluorescence of collagen I labelled with Texas Red C_2_-maleimide was significantly higher on all boron-doped NCD films than on the undoped films. The highest average intensity was obtained on NCD doped with 6700 ppm of B, where this value significantly exceeded that on NCD with 1000 ppm of B (**Fig. 10A**). The biochemical analysis (performed using high performance liquid chromatography combined with time of flight mass spectrometry) confirmed these findings, at least partly. Among the NCD samples, the highest amount of collagen was found on NCD with 6700 ppm of B, where this amount did not differ significantly from the value obtained on polystyrene. At the same time, the values on all remaining NCD samples (i.e., undoped and doped with 133 or 1000 ppm) were significantly lower than the value on polystyrene (**Fig. 10B**).

## Discussion

### Material and electronic properties of NCD thin films

The morphology of the doped and intrinsic nanocrystalline diamond films is comparable, in spite of slight variations in crystal sizes, shapes and surface roughness. The structural features are on the scale of tens of nanometres. The AFM phase measurements indicated high material quality of the NCD film surface. The contact angle values confirmed the comparable hydrophilic character of the NCD film surfaces. Raman spectroscopy confirmed overall high diamond quality of the diamond films. Only in the case of the 6700 ppm sample, which is close to metallic-like doping, did the non-diamond phase start to increase due to increasing disorder induced by the high doping level. Pronounced differences in the room temperature electrical resistivity of the NCD samples correlate well with the B-doping level, as expected. In spite of orders of magnitude differences in resistivity, the surface potentials remained about the same from the physical point of view. The observed spatial fluctuations of the surface potential can be mostly attributed to the surface roughness of the NCD samples.

### Supportive effects of NCD films on cell adhesion and osteogenic cell differentiation

We found that all NCD films investigated in our present study showed positive effects on the adhesion, viability and proliferation of human osteoblast-like MG 63 cells. Similar results were obtained in our earlier studies performed on MG 63 cells [19], [26]–[28], on another human-bone derived cell line SaOS-2 [48], and also on vascular endothelial cells [26] in cultures on oxygen-terminated nanostructured and hierarchically submicron-nanostructured NCD films. A variety of cells of other phenotypes, such as neuroblastoma cells, keratinocytes, macrophages and pheochromocytoma P12 cells, in cultures on NCD films or exposed to diamond nanoparticle suspensions (concentration of 5 to 100 µg per ml of the culture media), also retained their typical morphology, metabolic activity and viability [24]. Also in studies *in vivo*, diamond nanoparticles mixed with hyaluronan and introduced into rabbit bone were harmless and caused no decrease in new bone tissue formation [49]. This excellent biocompatibility of diamond nanoparticles can be explained by their high stability and inertness in comparison with other carbon nanoparticles, particularly fullerenes and nanotubes. The reactivity and the potential harmful effects of these nanoparticles are due to the presence of double bonds and bending of sp^2^-hybridized carbon atoms in the nanoparticle molecules, which produces angle strain. Fullerenes and nanotubes can convert molecular oxygen into highly reactive singlet oxygen. Thus, they have the potential to inflict damage on biological systems, including damage to cellular membranes, inhibition of various enzymes, DNA cleavage and thus genotoxic effects (for a review see [22]). By contrast, diamond nanoparticles were found to generate no significant amount of reactive oxygen species [24], [25]. Some authors have reported damaging effects of diamond nanoparticles on white and red blood cells *in vitro* and *in vivo* [50], [51]. However, in these studies, diamond nanoparticles were used in relatively high concentrations (1 mg/ml or more), whereas at lower concentrations (100 µg/ml) harmful effects were not observed. Also the size of the diamond particles was important for their potential damaging effects on cells. In cultures of HeLa cells, micro-sized diamond crystals (diameter approx. 1 µm, concentration ≥1 µm/ml) caused considerable stress to the cells and attenuated their growth and viability, while nanodiamond nanocrystals (diameter ∼2 nm) showed no harmful effects in comparable concentrations [52].

The cytotoxic action of carbon nanoparticles in general is associated with suspending them in the cell culture medium [53]. However, in our study, diamond nanoparticles were used only as the initial seeding layer on Si substrates for further growth of continuous and compact NCD thin films. Our earlier study showed that NCD-covered silicon substrates acted as excellent substrates for the adhesion, growth and viability of MG 63 cells, but without NCD films silicon substrates had significant cytotoxic effects on MG 63 cells [26]. In other words, our earlier study proved that our NCD films were continuous and covered the underlying substrate hermetically, and were mechanically and chemically stable in a cell culture environment. Thus, the release of diamond nanoparticles from our NCD films can be considered highly improbable.

Differentiation of MG 63 cells towards osteogenic phenotype was also enhanced on all NCD films in our study, particularly those doped with boron. It was indicated (1) that there were higher concentrations of osteocalcin in cells on these films compared to the values in cells on polystyrene dishes, (2) there was a higher concentration of alkaline phosphatase, i.e., an enzyme participating in bone tissue mineralization, in cells on NCD films doped with 1000 or 6700 ppm of B than in cells on the other samples, and also (3) that there was a higher concentration of collagen I, which is an important component of the bone matrix, in cells on the films with 133 ppm of B than in cells on undoped films and polystyrene. Similarly, in an earlier study [25], MG 63 cells and human bone marrow cells in cultures on NCD coatings deposited on silicon nitride substrates showed significantly higher activity of alkaline phosphatase than control cells on standard polystyrene cell culture dishes.

On the other hand, although the increase in the concentration of markers of osteogenic cell differentiation in cells on boron-doped NCD films compared to undoped samples was statistically significant, the absolute values were relatively small, usually reaching 16% to 33% (only the increase in concentration of alkaline phosphatase in cells on films doped with 1000 ppm of B amounted to 85%). This may be due to the fact that the cell lines, particularly those of tumor origin, including MG 63 cells, at least partly lose their markers of differentiation compared to primocultured or low-passaged cells [54]. In addition, the differentiation markers in our study were tested relatively early, i.e. 7 days after seeding. When the cultivation time is extended, more pronounced osteogenic cell differentiation can be expected. For example, Amaral *et al.* (2008) [25] reported maximum activity of alkaline phosphatase in bone marrow cells on NCD films on day 14 after seeding. Cell osteogenic differentiation could be further enhanced by functionalizing NCD with bone morphogenetic protein-2 (BMP-2). Titanium implants coated with O-terminated NCD layers modified by BMP-2 physisorption supported new bone tissue formation *in vivo* when implanted into sheep frontal bone [55].

Nevertheless, as shown in our earlier studies and in studies by other authors, even weak changes in the production of adhesion, cytoskeletal, differentiation and immunomodulatory molecules can significantly influence the morphology and behaviour of cells, e.g., vascular smooth muscle cells on collagen III substrates treated by UV light [56]. Similarly, in a study performed by Jeng *et al.* (1993) [57] on cultured human umbilical vein endothelial cells, even a small increase in the expression of immunoglobuline and selectin adhesion molecules, induced by treatment with oxidized human plasma low density lipids (LDL), resulted in a significant increase in the number and adhesion strength of monocytes bound to the endothelial cells.

The concentration of osteopontin, another ECM molecule considered as a marker of osteogenic cell differentiation, was similar on all tested samples. However, it should be pointed out that unlike osteocalcin, which is osteoblast-specific, osteopontin can also be synthesized by a wide spectrum of other cell types, e.g. vascular endothelial cells, smooth muscle cells, inflammatory cells [58], [59], chondrocytes [60] or skeletal muscle myoblasts [61]. The secretion of osteopontin is often enhanced under pathological conditions, such as cardiovascular, inflammatory, autoimmune and tumor diseases [58], [62], [63]. Osteopontin also facilitates bone resorption and remodelling [63]. Thus, osteopontin can be considered as a less typical indicator of physiological bone tissue formation than osteocalcin. In addition, like immunoglobulin and selectin adhesion molecules, osteopontin is also a marker of immune activation of cells [58], [62]. In accordance with the unchanged concentrations of osteopontin in cells on boron-doped NCD found in our study, the concentration of ICAM-1, an immunoglobulin adhesion molecule binding inflammatory cells, also remained unchanged. These findings suggest that none of the tested boron-doped NCD films evoked immune activation of MG 63 cells to a greater extent than in the conventional cell culture system on polystyrene dishes (**Fig. 9**). Similarly, in cultures of monocytes, diamond particles did not significantly increase the inflammatory response of these cells, evaluated by their production of interleukin-1. On the other hand, when exposed to particles of hydroxyapatite and silicon carbide, monocytes increased their interleukin-1 production on an average 30-fold and 38-fold, respectively, compared to control cultures without particles [64].

### Beneficial effects of surface nanoroughness on cell colonization and differentiation

The beneficial effect of both doped and undoped NCD films on the adhesion, growth and phenotypic maturation of MG 63 cells (compared to cells on standard polystyrene dishes) can be explained by their surface nanoroughness. The cell adhesion is mediated by ECM molecules, such as vitronectin, fibronectin, collagen and laminin, adsorbed to the material surface from biological fluids or deposited by cells. In our study, the adsorption of collagen I was relatively high on polystyrene, where it was higher or similar in comparison with all tested NCD films (**Fig. 10**). This result may be explained by the fact that commercially available tissue culture polystyrene is pre-activated by a glow discharge. In synthetic polymers, this and related treatments (e.g., irradiation with plasma, ions or UV light) lead to the creation of radicals and oxygen-containing groups which can bind various biomolecules (for a review, see [13]). The good affinity of polystyrene for biomolecules may account for the increase in cell number on polystyrene from day 3 to 7. While on day 3 the cell number on polystyrene was at least twice lower than on NCD films, on day 7 it was comparable with the values on undoped NCD and on NCD doped with 6700 ppm of B. The biomolecules are adsorbed to materials not only from the culture medium but they can be synthesized and deposited on the material surface by the cells themselves, which can mask the original physical and chemical properties of the material surface, and consequently the original differences in the cell behaviour.

On the other hand, it is generally known that not only the absolute number but also the geometrical conformation of the adsorbed cell adhesion-mediating molecules is important. It is believed that on nanostructured substrates, these molecules are adsorbed in a more physiological geometrical conformation, which enables better accessibility of specific bioactive sites in the ECM molecules (e.g., the Arg-Gly-Asp sequence and other adhesion oligopeptides) by cell adhesion receptors (e.g., integrins). In addition, nanostructured substrates have been reported to adsorb preferentially vitronectin (due to its relatively small, linear and less complicated molecule compared to other ECM molecules), which is recognized mainly by osteoblasts [6]–[8]. The positive effect of the surface nanostructure on cell colonization was probably further supported by the oxygen-termination and hydrophilicity of the NCD films used in our study (contact angle 14–20°). These factors are known to promote adsorption of cell adhesion-mediating molecules in a flexible conformation accessible by cell adhesion receptors, and thus to act synergetically with surface nanoroughness [12], [18], [33], [38], [42]; for a review, see [13], [22]).

### Electrical activity of the cell adhesion substrate

Another important factor supporting cell adhesion, growth and differentiation is the electroactivity of the cell adhesion substrate, e.g. its electrical charge, electrical potential and particularly its electrical conductivity. Electrical conductivity has been reported to act positively on cell performance even without additional electrical stimulation of the cells. For example, Schwann cells cultured on electrically conductive melanin films accelerated their proliferation, and PC12 cells enhanced the extension of their neurites [65]. Similarly, composite nanofibers made of electrically conductive polyaniline blended with poly(L-lactide-co-epsilon-caprolactone enhanced the adhesion and proliferation of C2C12 murine skeletal muscle myoblasts, as well as their differentiation towards myotubes [66], [67]. Also in studies *in vivo*, an electrically conductive polymer polypyrrole blended with alginate and injected into the infarcted zone of the heart in an ischemia-reperfusion rat myocardial infarction model supported neovascularization of the infarct area and its infiltration with myofibroblasts [68]. Electrically conductive materials also had a stimulatory effect on the adhesion, growth and phenotypic maturation of non-excitable cells, such as vascular endothelial cells [67], [68], fibroblasts [67], [69] and also bone cells [70]. Three-dimensional foam-like polyurethane scaffolds, the pore walls of which were decorated with electrically conductive multiwall carbon nanotubes, supported the attachment, growth, metabolic activity, mineralization and production of vascular endothelial growth factor in human osteoblast-like osteosarcoma SaOS-2 cells cultured on these scaffolds [70].

The mechanisms of the positive effects of electroactive materials on cell colonization and function (which can be further enhanced by additional electrical stimulation of cells by the materials) have not yet been fully elucidated and systemized. These mechanisms probably include enhanced adsorption of cell adhesion-mediating ECM proteins from biological environments, a more advantageous geometrical conformation of these proteins for their accessibility by cell adhesion receptors, redistribution of cell membrane growth factors and adhesion receptors or cytoskeletal proteins such as actin, activation of ion channels in the cell membrane, followed by cell depolarization, hyperpolarization or generation of action potential, movement of charged molecules inside and outside the cell, upregulated mitochondrial activity and enhanced protein synthesis (for a review, see [71]–[74]).

The electrical properties of boron-doped NCD layers in our study, manifested particularly by changes in electrical resistance (i.e., increasing electrical conductivity), may also explain the greater adsorption of collagen I on these films than on the undoped films (**Fig. 10**), enhanced adhesion of MG 63 cells on these films (indicated by better developed talin-containing focal adhesion plaques and a higher concentration of vinculin), faster proliferation of MG 63 cells on boron-doped films as well as more pronounced osteogenic differentiation of these cells, manifested by higher concentrations of osteocalcin, alkaline phosphatase and collagen I in cells on these films (**Fig. 9**). In an earlier study, another factor contributing to the better performance of SaOS-2 osteoblasts on NCD films was increased *rms* surface roughness. The metabolic activity of SaOS-2 osteoblasts, measured by the activity of dehydrogenases in these cells, increased with increasing surface roughness of O-terminated NCD films (*rms* from 11 to 39 nm) [48]. However, in our present study, no significant changes in the *rms* surface roughness were found. Direct action of boron on the cell metabolism is also less probable, because boron atoms are strongly bound in the NCD lattice or around the grain boundaries, and their release is very limited, even at very high temperatures combined with a vacuum and a strong electrical field, i.e. conditions not occurring in biological environments (for a review, see [42]).

### Different behaviour of cells on NCD films with various levels of B doping

Interestingly, the highest numbers of MG 63 cells were obtained on NCD substrates with low and medium B concentrations (133 and 1000 ppm, respectively), whereas on NCD with the highest B concentration (6700 ppm), the cell numbers declined. At the same time, the cells on NCD with 6700 ppm of B developed large and numerous focal adhesion plaques containing talin, and contained the highest concentration of focal adhesion protein vinculin. Both features are indicators of firm cell-substrate adhesion, which was probably enabled by the flattest appearance of the nanodiamond crystals on films doped with 6700 ppm of B (**Fig. 1**). These surfaces also adsorbed the highest amount of collagen I, an important cell adhesion-mediating ECM protein, as suggested by the fluorescence and biochemical evaluation (**Fig. 10**). It has been reported that cell proliferation activity is highest at intermediate cell-substrate adhesion strength (for a review, see [13]). If this strength is relatively high, the cells slow down their proliferation activity and enter the differentiation program. In accordance with this, the cells on NCD films with 6700 ppm of B contained one of the highest concentrations of osteocalcin and alkaline phosphatase, i.e., important markers of osteogenic cell differentiation, which were significantly higher than the values in cells on undoped NCD and on the control polystyrene dish. Similar results were also obtained in our earlier study performed on NCD films doped with 3000 ppm of B, where MG 63 cells were less numerous on boron-doped NCD films than on undoped NCD films, but they were more intensely immunofluorescently stained against osteocalcin [42].

In addition, the relatively low proliferation activity of cells on the samples doped with the highest B level may be correlated with the fact that these films exhibited a higher amount of sp^2^ bonded carbon (i.e. non-diamond phases), as observed by Raman spectroscopy. It was previously shown by AFM phase measurements that the standard surface wet chemical treatment may not remove all non-diamond phases from the surface [43]. These non-diamond phases with sp^2^ bonds typically include graphite or amorphous hydrogenated carbon (a-C∶H), i.e. materials, which often behave as bioinert and thus not promoting cell growth (for a review, see [13], [18], [22]).

Finally, the decline in cell number on samples with 6700 ppm of B could be attributed to the relatively high hydrophilicity of this material, indicated by the lowest water drop contact angle (13°) among all the B-doped samples. It is known that cell adhesion is optimum on moderately hydrophilic surfaces. Highly hydrophilic substrates bind the cell adhesion-mediating ECM molecules with relatively weak forces [13], [16], which can lead to the detachment of these molecules especially at later culture intervals, when they bind a large number of cells. In accordance with this, in our earlier study [27], a significant spontaneous cell loss of MG 63 cells (even if they were highly viable) was observed on day 5 after seeding of these cells on O-terminated NCD surfaces with a contact angle of 30°, while on H-terminated films with a contact angle of 85–90° the cells remained stably attached to the material surface [42], Similarly, in a study performed on extremely hydrophilic O-terminated nanostructured diamond surfaces (contact angle only 2°), these surfaces almost completely resisted even the initial adhesion of human mesenchymal stem cells derived from the bone marrow, whereas less hydrophilic nanodiamond surfaces (contact angle 86°) gave good support for the attachment, spreading and growth of these cells [75]. However, it should be pointed out that the differences in contact angles in these studies amounted to several tens, while in our present study, the differences in contact angle among the materials was much smaller. Their contact angle ranged from 14° to 20°, thus all of them can be classified as highly hydrophilic. From this point of view, no significant impact of the differences among their contact angles on the cell behaviour can be expected.

The electrical surface potentials on all samples remained within the range of ∼60 mV (**Tab. 1**). The observed spatial fluctuations of the surface potential can mostly be attributed to the surface roughness of the NCD samples. No abrupt border between the sp^2^ carbon phases localized at the grain boundaries and the grains consisting of sp^3^ was observed, in agreement with previous reports [76]. Nevertheless, biological experiments revealed that while the concentration of vinculin, osteocalcin and alkaline phosphatase, and also the adsorption of collagen I, were positively correlated rather with an increase in boron doping, i.e. the electrical conductivity of the material, the production of collagen I was associated with an increase in the surface potential. The concentration of collagen I in MG 63 cells reached maximum values on NCD films doped with 133 ppm of B, i.e. on the films with the highest surface potential (Table 1). More positive potential values correspond to a higher surface work function or to a more negative surface charge. Similar results were obtained in a recent study [36] performed on rabbit chondrocytes cultured on hydrogels. The production of collagen II and glycosaminoglycans, i.e. markers of chondrogenic cell differentiation, in these cells was positively correlated with an increase in the zeta potential of the material and with its negative charge.

### Conclusions and further perspectives

We found that both the intrinsic NCD films and the boron-doped NCD films accelerated the proliferation of human osteoblast-like MG 63 cells in cultures on these films in the early exponential phase of growth, increased their numbers and enhanced their differentiation towards the osteogenic cell phenotype, manifested by increased concentration of osteocalcin and collagen I per mg of cellular protein in comparison with cells grown on standard polystyrene dishes. These beneficial effects can be explained by the nanoroughness of NCD films and their wettability, and they were further enhanced by boron doping of these films. In cells on films doped with medium and high concentrations of boron, the concentration of alkaline phosphatase also significantly exceeded the value in cells on undoped films. These findings can be attributed to the electroactivity (i.e., electrical conductivity and surface electrical potential) of boron-doped NCD films. The increase in the cell number and proliferation activity was most apparent on semiconducting-like NCD films with 133 and 1000 ppm of B. NCD films with the highest B concentration (6×10^20^ cm^−3^ at 6700 ppm) supported mainly cell-matrix adhesion (manifested by the highest concentration of focal adhesion protein vinculin and increased adsorption of collagen I), and also osteogenic cell differentiation (suggested by a higher concentration of osteocalcin and alkaline phosphatase). At the same time, measurements of the concentration of ICAM-1 suggested that none of the tested NCD films evoked increased immune activation of MG 63 cells. Therefore, the NCD films constructed in this study are promising for coating bone implants in order to enhance their integration with the surrounding bone tissue. This integration may be further enhanced by electrical stimulation of osteoblasts through electrically-conductive boron-doped films.

## Materials and Methods

### Preparation of NCD films

Diamond thin films were deposited on SiO_2_ (1.4 µm)/Si(550 µm) substrates 10×10 mm^2^ in size or on Si substrates (5 cm in diameter). Ultrasonic treatment in a suspension of deionized water with fine-grained diamond powder (NanoAmando, New Metals and Chemicals Corp. Ltd., Kyobashi) was used as the seeding technique. The seeded substrates were loaded into the CVD chamber. CVD deposition was performed in a microwave plasma-enhanced CVD reactor using a three-step procedure: a) growth of the intrinsic diamond thin film, b) growth of boron-doped films, and c) growth of intrinsic diamond from the rear side of the substrate.

Deposition of intrinsic diamond was performed in the Aixtron P6 system with the following process parameters: total gas pressure 50 mbar, microwave power 2500 W, 1% of methane diluted in hydrogen, substrate temperature ∼950°C, growth time 3 hours (total film thickness 600 nm).

The boron-doped films were grown in the ASTeX 6500 system with the following parameters: total gas pressure 64 mbar, microwave power 4000 W, 3% of methane diluted in hydrogen, substrate temperature 800°C, growth time 18 minutes. Boron doping was achieved by adding trimethylboron (TMB) to the gas mixture, and the B∶C ratio was varied from 133 to 6700 ppm [43].

After deposition, all samples were chemically cleaned in a strong oxidizing mixture of hot sulphuric acid with potassium nitride (200°C, 30 minutes), followed by washing in deionized water for 20 minutes and drying with a nitrogen gun.

The hydrophilic character of the NCD surfaces was achieved by treatment with radio-frequency oxygen plasma (3 min, 300 W). The thickness of the boron-doped NCD films was about 350 nm in all cases [43].

### Characterization of the physical and chemical properties of NCD films

***The surface morphology*** of the grown films was investigated using field emission gun scanning electron microscopy (FESEM) operating in the secondary electron mode (e_Line, Raith).

***The diamond character*** of the grown structures was confirmed by Raman spectroscopy (Renishaw, Ramanscope 1000 microscope with Ar^+^ excitation wavelength 514.5 nm).

***The boron depth profiles*** were obtained by Neutron Depth Profiling (NDP). The NDP technique, a nuclear analytical technique, was used utilizing a strong nuclear reaction of 10B(n,alpha)7Li with a high cross section (3837 b). The detection limit and the depth resolution of the NDP technique depends on the intensity of the neutron beam, the Cd ratio (the purity of the thermal neutron beam) and the whole experimental set-up of the NDP spectrometer. For the NDP system used in this study, the detection limit for boron was about 10^12^ at.B/cm^2^, and the depth resolution of the technique was about 15 nm.

***The surface corrugation*** of the NCD films was characterized by atomic force microscopy (AFM) in a semicontact (vibrational) regime. The free cantilever amplitude was about 100 nm and the set-point ratio was about 50%, which provides optimal topography as well as phase contrast [77], [78]. Silicon cantilevers coated with a Pt/Cr layer were used. Their resonance frequency was 75 kHz and the nominal tip radius was 20 nm. The scans were performed and the root-mean-square (*rms*) surface roughness was calculated across the area of 1.5×1.5 µm^2^, which is much larger than a typical NCD grain size.

***The surface potentials*** of the boron-doped NCD films were characterized by Kelvin force microscopy (KFM). KFM was performed in a lift-mode, using the 2^nd^ pass technique [79]. The lift height for KFM was 30 nm. The measurements were made under ambient illumination. The metal coating on the AFM tips prevents photovoltage effects on the AFM tips. An a.c. excitation voltage 5 V in amplitude was applied to the tip. The samples were grounded via a spring contact on the top surface.

On highly resistive surfaces, such as the undoped NCD sample with an oxidized surface, KFM is detected against the floating potential of the surface. Nevertheless, such KFM measurement is still possible because the probing voltage is applied to the conducting tip [79], [80]. To compare the surface potential values among the NCD samples, a 50 nm gold layer was thermally evaporated on each sample and used as a reference electrode (grounded) for the KFM measurements. Positive potential values thus correspond to a higher surface work function or to a more negative surface charge. The gold layer had a work function of 5.0±0.1 eV, as determined by photoelectron spectroscopy after degassing in a vacuum. A lower work function 4.7±0.1 eV was obtained when the gold layer was not degassed, i.e. under similar conditions to KFM in air.

***The electrical resistivity measurements*** of the NCD samples were provided at room temperature by four-point contact mode using an Agilent digital multimeter. The measurement accuracy was better than 1%.

***The contact angle*** on the films was estimated using reflection goniometry using the Surface Energy Evaluation (SEE) System (Masaryk University, Brno, Czech Republic). Three µl of deionized water were dispensed on the NCD surfaces, and the formed drop was captured by a digital camera. The contact angle was calculated by SEE software using multipoint fitting of the drop profile and calculation of the tangent of the arc made by the drop.

### Cells and culture conditions

Square samples 10×10 mm^2^ in size or round samples (50 mm in diameter) were sterilized with 70% ethanol for 1 h. The smaller samples were placed in 24-well polystyrene multidishes (diameter 1.5 cm; TPP, Trasadingen, Switzerland) and were used for evaluating the cell number, morphology, viability and immunofluorescence staining of talin and beta-actin. The larger samples were inserted into polystyrene Petri dishes (GAMA GROUP a.s., Ceske Budejovice, Czech Republic; diameter 50 mm) and were used for enzyme-linked immunosorbent assays (ELISA) of molecular markers of cell adhesion, osteogenic differentiation and immune activation. Both types of samples were seeded with human osteoblast-like MG 63 cells (European Collection of Cell Cultures, Salisbury, UK). The cells were cultured in Dulbecco's modified Eagle's Minimum Essential Medium (DMEM; Sigma, Cat. No. D5648) supplemented with 10% fetal bovine serum (FBS; Gibco, Cat. No. 10270-106) and gentamicin (40 µg/ml, LEK, Slovenia) in a humidified air atmosphere containing 5% of CO_2_. The wells with smaller samples contained 20,000 cells (i.e., about 11,000 cells/cm^2^) and 1 ml of the culture medium, while the dishes with larger samples contained 200,000 cells/well (i.e., about 10,000 cells/cm^2^) and 6 ml of the medium.

### Evaluation of cell number, viability, spreading and growth dynamics

On day 1 and 3 after seeding, the cells on the samples were stained with the LIVE/DEAD viability/cytotoxicity kit for mammalian cells (Invitrogen, Molecular Probes, USA) according the manufacturer's protocol. Briefly, the cells were washed with phosphate-buffered saline (PBS; Sigma, Cat. No. P4417) and incubated in two probes detecting the esterase activity in living cells (calcein AM producing green fluorescence) or membrane damage in dead cells (ethidium homodimer-1 emitting red fluorescence) for 5 to 10 min at room temperature (∼25°C). Live and dead cells were then counted on microphotographs taken under an Olympus IX 51 epifluorescence microscope (obj. 20×) equipped with a DP 70 digital camera. Each picture represented an area of 0.136 mm^2^ on the sample.

On day 3 after seeding, the size of the cell spreading area (i.e., the cell area projected on to the material) was also measured in cells stained against cytoskeletal protein β-actin, using Atlas software (Tescan Ltd., Brno, Czech Republic). Cells entering into cell-cell contact, which may influence the size of the cell spreading area, were excluded from the evaluation.

On day 7 after seeding, when they were usually confluent and overlapping, the cells were counted in a Bürker haemocytometer. For this analysis, the cells were rinsed with PBS and released from the material with a trypsin-EDTA solution (Sigma, Cat. No. T4174; incubation 5 minutes at 37°C).

For the evaluation of the cell number and the size of the cell adhesion area, three independent experiments were performed in duplicates. On day 1 and 3, 16 pictures were taken in randomly selected fields homogeneously distributed on the surface of each sample (i.e., 32 pictures for each experimental group and time interval). On the 7^th^ day, 9 measurements in the Bürker haemocytometer were performed for each sample (i.e., 18 measurements for each experimental group).

The cell numbers obtained on days 1, 3 and 7 after seeding were used for constructing the growth curves and for calculating the cell population doubling time, using the following equation:
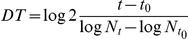
where *t_0_* and t represent earlier and later time intervals after seeding, respectively, and *N_t0_* and *N_t_* represent the number of cells at these intervals.

### Immunofluorescence staining of focal adhesion protein talin and cytoskeletal protein beta-actin

On day 3 after seeding, the cells were rinsed with PBS (Sigma) and fixed with 70% cold ethanol (−20°C, 5 min). Before exposure to the primary antibodies, the cells were pre-treated with 1% bovine serum albumin in PBS containing 0.1% Triton X-100 (Sigma) for 20 min at room temperature in order to block non-specific binding sites and to permeabilize the cell membrane. Mouse monoclonal anti-chicken talin (dilution 1∶200, Sigma, Cat. No. T3287, clone 8D4) and mouse monoclonal anti-β-actin (dilution 1∶200, Sigma, Cat. No. A5441, clone AC-15) were used as the primary antibodies. The antibodies were diluted in PBS at a concentration of 1∶200 and applied overnight at 4°C. After rinsing with PBS, the cells were incubated with the secondary antibody, namely goat anti-mouse F(ab′)_2_ fragment of IgG (dilution 1∶1000), conjugated with Alexa Fluor® 488 (Molecular Probes, Invitrogen; Cat. No. A11017), for 1 hour at room temperature and in a dark place. The cells were then rinsed twice in PBS, mounted under microscopic glass coverslips in Gel/Mount permanent fluorescence-preserving aqueous mounting medium (Biomeda Corporation, Foster City, CA, U.S.A.) and evaluated under an epifluorescence microscope (IX 51) equipped with a digital camera (DP 70, both from Olympus, Japan).

### Enzyme-linked immunosorbent assay (ELISA)

The concentrations of β_1_-integrin adhesion molecules (receptors for ECM molecules, mainly collagen and fibronectin), talin and vinculin (integrin-associated structural proteins of focal adhesion plaques), osteocalcin and osteopontin (ECM glycoproteins, markers of osteogenic cell differentiation), and immunoglobuline adhesion molecule ICAM-1 (CD 54, a marker of cell immune activation), were measured in homogenates of MG 63 cells after 7-day cultivation. The cells (10^6^ cells/ml), resuspended in distilled and deionized water, were kept in a freezer at −70°C overnight. The cells were then homogenized by ultrasonication for 40 seconds in a sonicator (UP 100 H, Dr. Hielscher GmbH) and the protein content was measured using a method originally developed by Lowry *et al.* [81] and modified by Filova *et al.* [15] and Grausova *et al.* [27]. Aliquots of the cell homogenates corresponding to 1–50 µg of protein in 50 µl of water were adsorbed on 96-well microtiter plates (Maxisorp, Nunc) at 4°C overnight. After washing twice with PBS (100 µl/well), the non-specific binding sites were blocked by 0.02% gelatin in PBS (100 µl/well, 60 min.) and then treated with 1% Tween (Sigma, Cat. No. P1379, 100 µl/well, 20 min). Primary antibodies, diluted in PBS and represented by monoclonal mouse anti-human β_1_-integrin (dilution 1∶200, Chemicon, Cat. No. MAB 1981, clone LM534), monoclonal mouse anti-chicken talin (dilution 1∶200, Sigma, Cat. No. T3287, clone 8D4), monoclonal mouse anti-human vinculin (dilution 1∶400, Sigma, Cat. No. V9131, clone hVIN-1), polyclonal rabbit anti-human osteopontin (dilution 1∶500, Alexis, Cat. No. ALX-210-309), monoclonal mouse anti-human placental alkaline phosphatase, (dilution 1∶200, Sigma, Cat. No. A 2951, clone 8B6), polyclonal rabbit anti-human osteocalcin (1–49) purified antiserum IgG (dilution 1∶500, Bachem Group, Peninsula Laboratories Inc., CA, USA; Cat. No. T-4743.0400), rabbit anti-bovine type I collagen, whole serum (dilution 1∶200, Cosmo Bio Co., Ltd., Cat. No. LSL-LB-1197), and monoclonal mouse anti-human ICAM-1 (anti CD-54) antibody (dilution 1∶200, Exbio s.r.o., CR; Cat. No. 11-228, clone MEM 111) were applied for 60 min. at room temperature (50 µl/well). As secondary antibodies, goat anti-mouse F(ab′)_2_ IgG fragment (Sigma, Cat. No. A3682, dilution 1∶1000) was used after mouse monoclonal primary antibodies, and goat anti-rabbit IgG (Sigma, Cat. No. A9169, dilution 1∶5000) was used after rabbit polyclonal antibodies. Both secondary antibodies were conjugated with peroxidase and applied for 45 min (100 µl/well). This step was followed by double washing in PBS and orthophenylendiamine reaction (Sigma, Cat. No. P1526, concentration 2.76 mM) using 0.05% H_2_O_2_ in 0.1 M phosphate buffer (pg 6.0, dark place, 100 µl/well). The reaction was stopped after 10–30 min by 2 M H_2_SO_4_ (50 µl/well), and the absorbance was measured at 490 and 690 nm by a Versa Max Microplate Reader (Molecular Devices Corporation, Sunnyvale, California, U.S.A). The absorbances obtained from cells growing on NCD films were expressed as a percentage of the values obtained in the control cultures on standard polystyrene dishes.

### Adsorption and quantification of collagen I

Collagen I derived from calf skin (Sigma, Cat. No. C8919, 0.1% solution in 0.1 M acetic acid) was diluted in distilled and deionized water to a concentration of 50 µg/ml. The sterilized NCD samples (10×10 mm) were placed into 24-well polystyrene multidishes (diameter 1.5 cm; TPP, Trasadingen, Switzerland), immersed in 0.5 ml of the collagen solution and incubated for 72 hours at 4°C. As a reference material, polystyrene wells were used. After incubation, the samples were rinsed three times with distilled water, and the amount of adsorbed collagen was then evaluated (1) by fluorescence microscopy and (2) by biochemical analysis, using High Performance Liquid Chromatography (HPLC) combined with Time of Flight Mass Spectrometry (TOF-MS).

#### Fluorescence microscopy

Collagen adsorbed on the studied samples was labelled with Texas Red C_2_-Maleimide (excitation maximum 595 nm, emission maximum 615 nm; Molecular Probes, Invitrogen, Cat. No. T6008) [82]. The dye was diluted in PBS to a concentration of 20 ng/ml, and the samples were incubated for 2 hours at room temperature in the dark. The samples were then rinsed three times with distilled water, and fluorescence pictures were taken under an Olympus IX 51 microscope equipped with a DP 70 digital camera (exposure time 5 s). For each experimental group, 2 samples were used, and from each sample, 5–8 pictures were taken. In the pictures, the intensity of the fluorescence was measured using the *Ellipse* modular software package (ViDiTo, Kosice, Slovakia; www.vidito.com). The intensity of the fluorescence was also measured on the control samples without exposure to collagen (i.e., exposed only to Texas Red C_2_-Maleimide), and this value was then subtracted from the corresponding samples adsorbed with collagen.

#### Biochemical analysis of collagen

The NCD samples after rinsing were transferred into vials, and were digested in 1 ml of a solution containing NH_4_HCO_3_ (0.05 mol/l) and trypsin (0.003 mg/ml) at 37°C overnight. All samples were lyophilized and dissolved in 25 µl of 0.1% acetic acid.

Chromatographic separation was carried out in an NS-AC-11-C18 Biosphere C18 analytical column (particle size: 5 µm, pore size: 12 nm, length 150 mm), and a NS-MP-10 Biosphere C18 precolumn (particle size: 5 µm, pore size: 12 nm, length 20 mm), both from NanoSeparations (Nieuwkoop, Netherlands).

The nano-HPLC apparatus used was Proxeon Easy-nLC (Proxeon, Odense, Denmark). It was coupled to a QTOF (quadrupole – time-of-flight) mass spectrometer with ultrahigh resolution (UHR-TOF) maXis (Bruker Daltonics, Bremen, Germany).

For the peptide identification and selection experiments, separation of peptides was achieved via a linear gradient between mobile phase A (water/formic acid, 100∶0.1 v/v) and B (ACN/formic acid, 100∶0.1 v/v). The water, acetonitrile and formic acid that were used were of LC-MS quality (Sigma-Aldrich, St. Louis, MO, USA). In addition to the trypsin (type IX-S, lot 51K72501), acetic acid and NH_4_HCO_3_ were obtained from Sigma-Aldrich.

Separation was started by running the system with 5% mobile phase B, followed by gradient elution to 30% B at 70 min. The next step was gradient elution to 50% B in 10 min, then a gradient to 100% B in 8 min. Finally, the column was eluted with 100% B for 2 min. Equilibration before the next run was achieved by washing with 5% mobile phase B for 10 min.

The flow-rate was 0.25 µl/min, the injection volume was 5 µl, and the column temperature was held at laboratory temperature (25°C). Nano-electrospray ionization (easy nano-ESI) in positive mode was used, and the ESI voltage was −4.5 kV. Operating conditions: drying gas (N_2_), 1 l/min; drying gas temperature, 160°C; nebulizer pressure, 0.4 bar. For the peptide selection experiments, ions were observed over the mass range m/z 100–2200.

Searches were performed in the SwissProt full protein database and then on the data extracted from this database. The ProteinScape 2 and ProfileAnalysis evaluation programs were used (both Bruker Daltonics, Bremen, Germany).

### Statistical analysis

The quantitative data on the physical and chemical properties of the material was presented as mean ± S.D. (Standard Deviation). The quantitative data obtained in the biological experiments was presented as mean ± S.E.M. (Standard Error of the Mean). The statistical analyses were performed using SigmaStat (Jandel Corporation, U.S.A.). The multiple comparison procedures were carried out by the ANOVA, Student-Newman-Keuls Method. The value *p*≤0.05 was considered significant.

## References

[1] Stevens MM, George JH (2005). Exploring and engineering the cell surface interface.. Science.

[2] Bacakova L, Stary V, Kofronova O, Lisa V (2001). Polishing and coating carbon fibre-reinforced carbon composites with a carbon-titanium layer enhances adhesion and growth of osteoblast-like MG63 cells and vascular smooth muscle cells *in vitro*.. J Biomed Mater Res.

[3] Stary V, Glogar P, Bacakova L, Hnilica F, Chmelik V (2003). A study of surface properties of composite materials and their influence on the biocompatibility.. Acta Montana AB.

[4] Kim HJ, Kim SH, Kim MS, Lee EJ, Oh HG (2005). Varying Ti-6Al-4V surface roughness induces different early morphologic and molecular responses in MG63 osteoblast-like cells.. J Biomed Mater Res.

[5] Sader MS, Balduino A, de Soares A, Borojevic R (2005). Effect of three distinct treatments of titanium surface on osteoblast attachment, proliferation, and differentiation.. Clin Oral Implants Res.

[6] Miller DC, Thapa A, Haberstroh KM, Webster TJ (2004). Endothelial and vascular smooth muscle cell function on poly(lactic-co-glycolic acid) with nano-structured surface features.. Biomaterials.

[7] Price RL, Ellison K, Haberstroh KM, Webster TJ (2004). Nanometer surface roughness increases select osteoblast adhesion on carbon nanofiber compacts.. J Biomed Mater Res A.

[8] Webster TJ, Ejiofor JU (2004). Increased osteoblast adhesion on nanophase metals: Ti, Ti6Al4V, and CoCrMo.. Biomaterials.

[9] Schmidt RC, Healy KE (2009). Controlling biological interfaces on the nanometer length scale.. J Biomed Mater Res A.

[10] Kim DH, Lee H, Lee YK, Nam JM, Levchenko A (2010). Biomimetic nanopatterns as enabling tools for analysis and control of live cells.. Adv Mater.

[11] Wong LS, Janusz SJ, Sun S, Leggett GJ, Micklefield J (2010). Nanoscale biomolecular structures on self-assembled monolayers generated from modular pegylated disulfides.. Chemistry.

[12] Svorcik V, Kasalkova N, Slepicka P, Zaruba K, Bacakova L (2009). Cytocompatibility of Ar^+^ plasma-treated and Au nanoparticle-grafted PE.. Nucl Instr Meth Phys Res B.

[13] Bacakova L, Svorcik V, Kimura D (2008). Cell colonization control by physical and chemical modification of materials.. Cell Growth Processes: New Research.

[14] Prabhakaran MP, Venugopal J, Ramakrishna S (2009). Electrospun nanostructured scaffolds for bone tissue engineering.. Acta Biomater.

[15] Filova E, Brynda E, Riedel T, Bacakova L, Chlupac J (2009). Vascular endothelial cells on two- and three-dimensional fibrin assemblies for biomaterial coatings.. J Biomed Mater Res.

[16] Bacakova L, Filova E, Kubies D, Machova L, Proks V (2007). Adhesion and growth of vascular smooth muscle cells in cultures on bioactive RGD peptide-carrying polylactides.. J Mater Sci Mater Med.

[17] Chen F, Lam WM, Lin CJ, Qiu GX, Wu ZH (2007). Biocompatibility of electrophoretical deposition of nanostructured hydroxyapatite coating on roughen titanium surface: in vitro evaluation using mesenchymal stem cells.. J Biomed Mater Res B Appl Biomater.

[18] Grinevich A, Bacakova L, Choukourov A, Boldyryeva H, Pihosh Y (2009). NanocompositeTi/hydrocarbon plasma polymer films from reactive magnetron sputtering as growth supports for osteoblast-like and endothelial cells.. J Biomed Mater Res.

[19] Bacakova L, Grausova L, Vacik J, Fraczek A, Blazewicz S (2007). Improved adhesion and growth of human osteoblast-like MG 63 cells on biomaterials modified with carbon nanoparticles.. Diamond Relat Mater.

[20] Liu H, Slamovich EB, Webster TJ (2006). Increased osteoblast functions among nanophase titania/poly(lactide-co-glycolide) composites of the highest nanometer surface roughness.. J Biomed Mater Res A.

[21] Hallab NJ, Jacobs JJ (2009). Biologic effects of implant debris.. Bull NYU Hosp Jt Dis.

[22] Bacakova L, Grausova L, Vandrovcova M, Vacik J, Frazcek A, Lombardi SL (2008). Carbon nanoparticles as substrates for cell adhesion and growth.. Nanoparticles: New Research.

[23] Rezek B, Kratka M, Ukraintsev E, Babchenko O, Kromka A, Serra PA (2011). Diamond as functional material for bioelectronics and biotechnology.. Biosensors for Health, Environment and Biosecurity.

[24] Schrand AM, Huang H, Carlson C, Schlager JJ, Osawa E (2007). Are diamond nanoparticles cytotoxic?. J Phys Chem B.

[25] Amaral M, Dias AG, Gomes PS, Lopes MA, Silva RF (2008). Nanocrystalline diamond: In vitro biocompatibility assessment by MG 63 and human bone marrow cells cultures.. J Biomater Res A.

[26] Grausova L, Kromka A, Bacakova L, Potocky S, Vanecek M (2008). Bone and vascular endothelial cells in cultures on nanocrystalline diamond films.. Diamond Relat Mater.

[27] Grausova L, Bacakova L, Kromka A, Vanecek M, Lisa V (2009). Molecular markers of adhesion, maturation and immune activation of human osteoblast-like MG 63 cells on nanocrystalline diamond films.. Diamond Relat Mater.

[28] Grausova L, Bacakova L, Kromka A, Potocky S, Vanecek M (2009). Nanodiamond as a promising material for bone tissue engineering.. J Nanosci Nanotechnol.

[29] Rupprecht S, Bloch A, Rosiwal S, Neukam FW, Wiltfang J (2002). Examination of the bone-metal interface of titanium implants coated by the microwave plasma chemical vapor deposition method.. Int J Oral Maxillofac Implants.

[30] Papo MJ, Catledge SA, Vohra YK, Machado C (2004). Mechanical wear behavior of nanocrystalline and multilayer diamond coatings on temporomandibular joint implants.. J Mater Sci Mater Med.

[31] Kong XL, Huang LCL, Hsu C-M, Chen W-H, Han C-C (2005). High-affinity capture of proteins by diamond nanoparticles for mass spectrometric analysis.. Anal Chem.

[32] Bondar VS, Pozdnyakova IO, Puzyr AP (2004). Applications of nanodiamonds for separation and purification of proteins.. Phys Solid State.

[33] Heitz J, Svorcik V, Bacakova L, Rockova K, Ratajova E (2003). Cell adhesion on polytetrafluoroethylene modified by UV-irradiation in an ammonia atmosphere.. J Biomed Mater Res A.

[34] Liu L, Chen S, Giachelli CM, Ratner BD, Jiang S (2005). Controlling osteopontin orientation on surfaces to modulate endothelial cell adhesion.. J Biomed Mater Res A.

[35] Lesny P, Pradny M, Jendelova P, Michalek J, Vacik J (2006). Macroporous hydrogels based on 2-hydroxyethyl methacrylate. Part 4: growth of rat bone marrow stromal cells in three-dimensional hydrogels with positive and negative surface charges and in polyelectrolyte complexes.. J Mater Sci Mater Med.

[36] Dadsetan M, Pumberger M, Casper ME, Shogren K, Giuliani M (2011). The effects of fixed electrical charge on chondrocyte behavior.. Acta Biomater.

[37] Svorcik V, Rybka V, Hnatowicz V, Bacakova L (1995). Polarity, resistivity and biocompatibility of polyethylene doped with carbon black.. J Mater Sci Lett.

[38] Svorcik V, Rybka V, Hnatowicz V, Bacakova L, Lisa V (1995). Surface properties and biocompatibility of ion implanted polymers.. J Mater Chem.

[39] Gajewski W, Achatz P, Williams OA, Haenen K, Bustarret E (2009). Electronic and optical properties of boron-doped nanocrystalline diamond films.. Phys Rev B.

[40] Nebel CE, Shin D, Rezek B, Tokuda N, Uetsuka H (2007). Diamond and biology.. J R Soc Interface.

[41] Marcon L, Spriet C, Coffinier Y, Galopin E, Rosnoblet C (2010). Cell adhesion properties on chemically micropatterned boron-doped diamond surfaces.. Langmuir.

[42] Kopecek M, Bacakova L, Vacik J, Fendrych F, Vorlicek V (2008). Improved adhesion, growth and maturation of human bone-derived cells on nanocrystalline diamond films.. Phys Stat Sol (a).

[43] Kromka A, Grausova L, Bacakova L, Vacik J, Rezek B (2010). Semiconducting to metallic-like boron doping of nanocrystalline diamond films and its effect on osteoblastic cells.. Diamond Relat Mater.

[44] Jacobs HO, Leuchtmann P, Homan OJ, Stemmer A (1998). Resolution and contrast in Kelvin probe force microscopy.. J Appl Phys.

[45] Kozak H, Kromka A, Ukraintsev E, Zemek J, Ledinsky M (2009). Detecting sp^2^ phase on diamond surfaces by atomic force microscopy phase imaging and its effects on surface conductivity.. Diamond Relat Mater.

[46] Gupta S, Williams OA, Bohannan E (2007). Electrostatic force microscopy studies of boron-doped diamond films.. J Mater Res.

[47] Ye H, Sun CQ, Hing P (2000). Control of grain size and size effect on the dielectric constant of diamond films.. J Phys D: Appl Phys.

[48] Kalbacova M, Kalbac M, Dunsch L, Kromka A, Vanecek M (2007). The effect of SWCNT and nano-diamond films on human osteoblast cells.. Phys Stat Sol (b).

[49] Aspenberg P, Anttila A, Konttinen YT, Lappalainen R, Goodman SB (1996). Benign response to particles of diamond and SiC: bone chamber studies of new joint replacement coating materials in rabbits.. Biomaterials.

[50] Puzyr' AP, Neshumaev DA, Tarskikh SV, Makarskaia GV, Dolmatov VI (2005). Destruction of human blood cells upon interaction with detonation nanodiamonds in experiments in vitro.. Biofizika.

[51] Karpukhin AV, Avkhacheva NV, Kulakova II, Yakovlev RY, Yashin VA (2010). Effect of detonation nanodiamonds on phagocyte activity.. Cell Biol Int.

[52] Fucikova A, Valenta J, Pelant I, Brezina V (2009). Novel use of silicon nanocrystals and nanodiamonds in biology.. Chem Pap.

[53] Yamawaki H, Iwai N (2006). Cytotoxicity of water-soluble fullerene in vascular endothelial cells.. Am J Physiol Cell Physiol.

[54] Tan J, Saltzman WM (2004). Biomaterials with hierarchically defined micro- and nanoscale structure.. Biomaterials.

[55] Kloss FR, Gassner R, Preiner J, Ebner A, Larsson K (2008). The role of oxygen termination of nanocrystalline diamond on immobilisation of BMP-2 and subsequent bone formation.. Biomaterials.

[56] Bacakova L, Lisa V, Kubinova L, Wilhelm J, Novotna J (2002). UV light - irradiated collagen III modulates expression of cytoskeletal and surface adhesion molecules in rat aortic smooth muscle cells *in vitro*.. Virchow's Archiv.

[57] Jeng JR, Chang CH, Shieh SM, Chiu HC (1993). Oxidized low-density lipoprotein enhances monocyte-endothelial cell binding against shear-stress-induced detachment.. Biochim Biophys Acta.

[58] Scatena M, Liaw L, Giachelli CM (2007). Osteopontin: a multifunctional molecule regulating chronic inflammation and vascular disease.. Arterioscler Thromb Vasc Biol.

[59] Duvall CL, Weiss D, Robinson ST, Alameddine FM, Guldberg RE (2008). The role of osteopontin in recovery from hind limb ischemia.. Arterioscler Thromb Vasc Biol.

[60] Gerstenfeld LC, Shapiro FD (2996). Expression of bone-specific genes by hypertrophic chondrocytes: implication of the complex functions of the hypertrophic chondrocyte during endochondral bone development.. J Cell Biochem.

[61] Uaesoontrachoon K, Yoo HJ, Tudor EM, Pike RN, Mackie EJ (2008). Osteopontin and skeletal muscle myoblasts: association with muscle regeneration and regulation of myoblast function in vitro.. Int J Biochem Cell Biol.

[62] Braitch M, Constantinescu CS (2010). The role of osteopontin in experimental autoimmune encephalomyelitis (EAE) and multiple sclerosis (MS).. Inflamm Allergy Drug Targets.

[63] Shevde LA, Das S, Clark DW, Samant RS (2010). Osteopontin: an effector and an effect of tumor metastasis.. Curr Mol Med.

[64] Nordsletten L, Høgåsen AKM, Konttinen YT, Santavirta S, Aspenberg P (1996). Human monocytes stimulation by particles of hydroxyapatite, silicon carbide and diamond: in vitro studies of new prosthesis coatings.. Biomaterials.

[65] Bettinger CJ, Bruggeman JP, Misra A, Borenstein JT, Langer R (2009). Biocompatibility of biodegradable semiconducting melanin films for nerve tissue engineering.. Biomaterials.

[66] Jeong SI, Jun ID, Choi MJ, Nho YC, Lee YM (2008). Development of electroactive and elastic nanofibers that contain polyaniline and poly(L-lactide-co-epsilon-caprolactone) for the control of cell adhesion.. Macromol Biosci.

[67] Jun I, Jeong S, Shin H (2009). The stimulation of myoblast differentiation by electrically conductive sub-micron fibers.. Biomaterials.

[68] Mihardja SS, Sievers RE, Lee RJ (2008). The effect of polypyrrole on arteriogenesis in an acute rat infarct model.. Biomaterials.

[69] Shi G, Zhang Z, Rouabhia M (2008). The regulation of cell functions electrically using biodegradable polypyrrole-polylactide conductors.. Biomaterials.

[70] Verdejo R, Jell G, Safinia L, Bismarck A, Stevens MM (2009). Reactive polyurethane carbon nanotube foams and their interactions with osteoblasts.. J Biomed Mater Res A.

[71] Schmidt CE, Shastri VR, Vacanti JP, Langer R (1997). Stimulation of neurite outgrowth using an electrically conducting polymer.. Proc Natl Acad Sci U S A.

[72] Gomez N, Schmidt CE (2007). Nerve growth factor-immobilized polypyrrole: bioactive electrically conducting polymer for enhanced neurite extension.. J Biomed Mater Res A.

[73] Khang D, Park GE, Webster TJ (2008). Enhanced chondrocyte densities on carbon nanotube composites: the combined role of nanosurface roughness and electrical stimulation.. J Biomed Mater Res A.

[74] Shi G, Rouabhia M, Meng S, Zhang Z (2008). Electrical stimulation enhances viability of human cutaneous fibroblasts on conductive biodegradable substrates.. J Biomed Mater Res A.

[75] Clem WC, Chowdhury S, Catledge SA, Weimer JJ, Shaikh FM (2008). Mesenchymal stem cell interaction with ultra-smooth nanostructured diamond for wear-resistant orthopaedic implants.. Biomaterials.

[76] Verveniotis E, Cermak J, Kromka A, Ledinsky M, Remes Z (2010). Local electrostatic charging differences of sub-100 nm nanocrystalline diamond films.. Phys Stat Sol (a).

[77] Garcia R, Gomez CJ, Martinez NF, Patil S, Dietz C (2006). Identification of nanoscale dissipation processes by dynamic atomic force microscopy.. Phys Rev Let.

[78] Rezek B, Shin D, Uetsuka H, Nebel CE (2007). Microscopic diagnostics of DNA molecules on mono-crystalline diamond.. Phys Stat Sol (a).

[79] Rezek B, Nebel CE, Stutzmann M (2004). Hydrogenated diamond surfaces studied by atomic and Kelvin force microscopy.. Diamond Relat Mater.

[80] Rezek B (2005). Atomic and Kelvin force microscopy applied on hydrogenated diamond surfaces.. New Diam Front C Tech.

[81] Lowry OH, Rosebrough NJ, Farr AL, Randall RJ (1951). Protein measurement with the Folin phenol reagent.. J Biol Chem.

[82] Langenbach KJ, Elliott JT, Tona A, McDaniel D, Plant AL (2006). Thin films of Type 1 collagen for cell by cell analysis of morphology and tenascin-C promoter activity.. BMC Biotechnology.

